# p300/CBP-associated factor promotes autophagic degradation of δ-catenin through acetylation and decreases prostate cancer tumorigenicity

**DOI:** 10.1038/s41598-019-40238-w

**Published:** 2019-03-04

**Authors:** Rui Zhou, Yi Yang, So-Yeon Park, Young-Woo Seo, Sang-Chul Jung, Kyung Keun Kim, Kwonseop Kim, Hangun Kim

**Affiliations:** 1 College of Pharmacy and Research Institute of Life and Pharmaceutical Sciences, Sunchon National University, Sunchon, Republic of Korea; 2 Korea Basic Science Institute, Gwangju Center, Gwangju, Republic of Korea; 3 Department of Environmental Engineering, Sunchon National University, Sunchon, Republic of Korea; 4 Department of Pharmacology, Chonnam National University Medical School, Gwangju, Republic of Korea; 5 College of Pharmacy and Research Institute for Drug Development, Chonnam National University, Gwangju, Republic of Korea

## Abstract

δ-Catenin shares common binding partners with β-catenin. As acetylation and deacetylation regulate β-catenin stability, we searched for histone acetyltransferases (HATs) or histone deacetylases (HDACs) affecting δ-catenin acetylation status and protein levels. We showed that p300/CBP-associated factor (PCAF) directly bound to and acetylated δ-catenin, whereas several class I and class II HDACs reversed this effect. Unlike β-catenin, δ-catenin was downregulated by PCAF-mediated acetylation and upregulated by HDAC-mediated deacetylation. The HDAC inhibitor trichostatin A attenuated HDAC1-mediated δ-catenin upregulation, whereas HAT or autophagy inhibitors, but not proteasome inhibitors, abolished PCAF-mediated δ-catenin downregulation. The results suggested that PCAF-mediated δ-catenin acetylation promotes its autophagic degradation in an Atg5/LC3-dependent manner. Deletions or point mutations identified several lysine residues in different δ-catenin domains involved in PCAF-mediated δ-catenin downregulation. PCAF overexpression in prostate cancer cells markedly reduced δ-catenin levels and suppressed cell growth and motility. PCAF-mediated δ-catenin downregulation inhibited E-cadherin processing and decreased the nuclear distribution of β-catenin, resulting in the suppression of β-catenin/LEF-1-mediated downstream effectors. These data demonstrate that PCAF downregulates δ-catenin by promoting its autophagic degradation and suppresses δ-catenin-mediated oncogenic signals.

## Introduction

The catenins (α, β, γ, δ, and p120) are cytoplasmic proteins which are related to the Drosophila Armadillo protein. β-Catenins are components of adherens junctional cadherin complex by bind to the cytoplasmic tail of E-cadherin and can transduce intracellular signal to the nucleus in the Wnt signaling pathway. The p120-catenin family (p120-catenin, δ-catenin, ARVCF, p0071, pkp2, and pkp3) is homologous to both β- and γ-catenin and is a substrate of tyrosine kinases with cadherin/catenin complex at adherens junctions^1^. δ-Catenin was identified by its association with Alzheimer’s disease-related protein presenilin-1^2^, and is most closely related to p120-catenin and the desmosomal protein p0071. Structurally, it contains 10 Armadillo (ARM) repeat domains, whereas β-catenin has 13 ARM repeat domains. Moreover, β- and δ-catenin conduce the adhesive potential of cadherin-based cell-cell contacts and share similar binding partners in signaling pathways including E-cadherin^3,4^. δ-Catenin promotes the fragmentation of E-cadherin (also known as E-cadherin processing), leading to increased total β-catenin protein levels and nuclear distribution, and resulting in the activation of β-catenin/LEF-1-mediated transcription^5^. These findings suggest that δ- and β-catenin are closely related and share similar signaling functions.

δ-Catenin is abundantly expressed in the developing neurons, which suggests the involvement of it in neuronal progenitor cell migration and dendrite development^6,7^. δ-Catenin is also overexpressed in various human malignancies, including prostate^3,8^, brain^9^, breast^10^, lung^11^, ovary^12^, esophagus^13^, and colorectal cancer^14^. In prostate cancer, δ-catenin accumulation promotes cancer cell growth and tumorigenesis by altering the cell cycle and the expression profiles of survival-related genes^8^. Furthermore, δ-catenin promotes prostate tumor growth by increasing angiogenesis through the upregulation of HIF-1α and VEGF^15^. Human prostate cancer cells overexpressing δ-catenin show an increase in multi-layer growth and substantial processing of plasma membranous E-cadherin, suggesting that δ-catenin plays a role in prostate cancer progression by inducing E-cadherin processing and thereby the release of β-catenin and increased oncogenic signaling^5^. Increased β-catenin translocates to the nucleus, where it functions in transcriptional regulation through interactions with transcription factors of the LEF-1/TCF family^16^.

Transcription is the first step in gene expression leading to the generation of a functional protein product^17^. Post-translational modifications such as phosphorylation, acetylation, methylation, and ubiquitination modulate the activity or stability of proteins^18,19^. The cellular protein degradation machinery includes the ubiquitin-proteasome pathway and the endosome-lysosome pathway, which control the degradation of the majority of eukaryotic proteins. We previously showed that δ-catenin is ubiquitinated and targeted for degradation by the ubiquitin-proteasome pathway^4^. However, the molecular mechanism of δ-catenin degradation mediated by the lysosomal pathway remains unknown.

To clarify the mechanisms underlying the regulation of δ-catenin and the maintenance of adequate δ-catenin protein levels in cells, we investigated δ-catenin stabilization through acetylation. Acetylation mostly results in protein stabilization, which is the case for β-catenin^20,21^ and regulatory T cells^22^. The acetyltransferase p300/CBP-associated factor (PCAF) catalyzes β-catenin acetylation and promotes its stability in cells^21^. PCAF is a transcription cofactor that possesses intrinsic histone acetyltransferase (HAT) activity^23^. PCAF-mediated acetylation affects different biological functions, such as transcriptional activity, stability, and subcellular localization. PCAF regulates p21 transcription by catalyzing the stress-induced acetylation of histone H3, and acetylates the tumor suppressor p53 in response to DNA damage^24,25^. In the present study, we show that PCAF acetylates and significantly downregulates δ-catenin by promoting its degradation via the autophagosomal pathway. Our results suggest that targeting acetylation may be an effective strategy in δ-catenin-associated carcinomas.

## Results

### PCAF acetylates and downregulates δ-catenin, whereas HDACs deacetylate and upregulate δ-catenin

**Figure 1:**
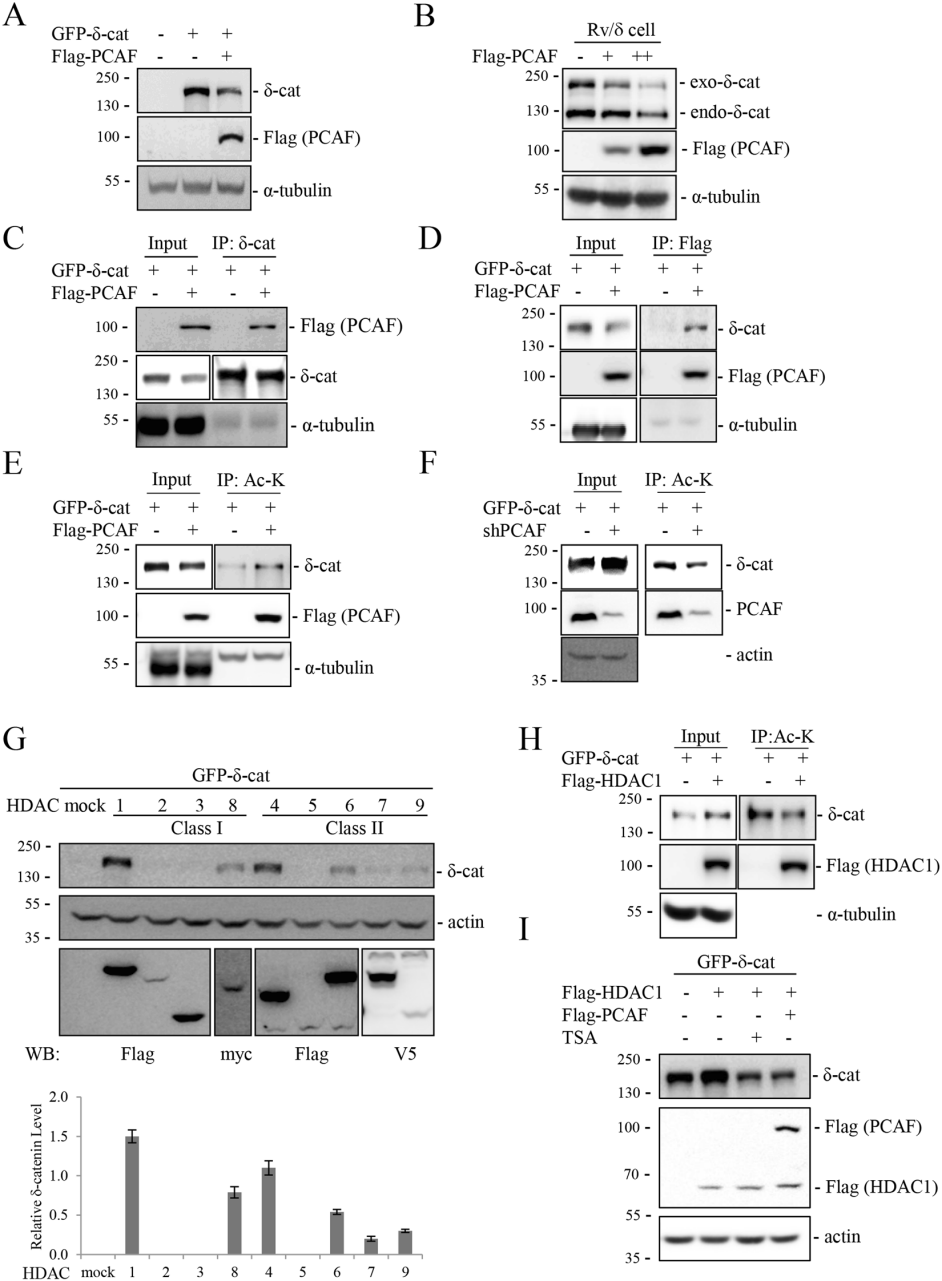
PCAF and HDACs regulate the acetylation status and levels of δ-catenin. (**A**,**B**) PCAF decreases δ-catenin levels. HEK293T (**A**) and δ-catenin-overexpressing CWR22Rv-1 (Rv/δ) (**B**) cells were transfected as indicated, and cell lysates were subjected to immunoblotting. (**C**–**F**) PCAF interacts with and acetylates δ-catenin. HEK293T cells were transfected as indicated, and cell lysates were subjected to immunoprecipitation with anti-δ-catenin (**C**), anti-Flag (**D**), or anti-acetylated-lysine (**E**,**F**), followed by immunoblotting of precipitated proteins. (**G**–**I**) HDACs deacetylate and upregulate δ-catenin. HEK293T cells were transfected as indicated, and cell lysates were subjected to immunoblotting (**G**) or immunoprecipitation with anti-acetylated-lysine (**H**). At 12 h post-transfection of HEK293T cells with GFP-δ-catenin and HDAC1, cells were treated with 0.2 µM trichostatin A (TSA) or transfected with Flag-PCAF, and incubated for 24 h, followed by immunoblotting with anti-δ-catenin and anti-Flag antibodies (**I**). α-Tubulin or ß-actin was used as a loading control. Relative δ-catenin/actin/HDACs ratios from three different experimental results are shown as a bar graph (**G**). Values are presented as the mean ± SEM. “−”, Mock transfection or vehicle treatment.

Acetylation is a post-translational modification that modulates the activity and/or amount of non-histone proteins. Acetylation of β-catenin at a Lys epsilon-amino group improves its stability by interfering with ubiquitination and thereby preventing its proteasomal degradation^21^. To determine whether acetylation is involved in the regulation of δ-catenin, several HAT enzymes including p300, PCAF, and MOZ were co-transfected with δ-catenin, and changes in the level of δ-catenin were analyzed by immunoblotting. Among the tested HAT enzymes, PCAF significantly downregulated δ-catenin, whereas p120-catenin remained unchanged (Fig. S1A); these results were confirmed in HEK293T cells and δ-catenin-overexpressing CWR22Rv-1 (Rv) prostate cancer cells (Rv/δ) (Fig. 1A,B). The interaction between δ-catenin and PCAF was confirmed by immunoprecipitation (Fig. 1C,D), which also demonstrated the increase in δ-catenin acetylation in response to co-transfection with PCAF (Fig. 1E). Conversely, knockdown of PCAF decreased the acetylation of and upregulated δ-catenin (Fig. 1F). p300 also downregulated δ-catenin to some extent, although a marginal induction of acetylation was observed (Fig. S1A,B). These results indicated that PCAF interacted with and acetylated δ-catenin, and, unlike its effect on β-catenin, the increased acetylation downregulated the expression of δ-catenin.

HDAC and HAT enzymes have opposing activities. To examine whether HDACs counteract the action of HATs on δ-catenin, the effect of class I and II HDACs on δ-catenin levels were examined. HDAC1, HDAC8 and HDAC4, which are class I and II HDACs, respectively, markedly upregulated δ-catenin (Fig. 1G). HDAC6, HDAC7, and HDAC9, which are class II HDACs, showed marginal effects on increasing δ-catenin levels. Transfection with HDAC1 was used to verify the HDAC-mediated decrease in the level of δ-catenin acetylation (Fig. 1H), and the HDAC1-induced upregulation of δ-catenin was restored by the HDAC inhibitor trichostatin A (TSA) or PCAF overexpression (Fig. 1I). Taken together, these results demonstrated that PCAF and HDACs regulate the acetylation status and levels of δ-catenin.

### PCAF-mediated δ-catenin downregulation is dependent on its acetyltransferase activity and cellular autophagy activity

**Figure 2:**
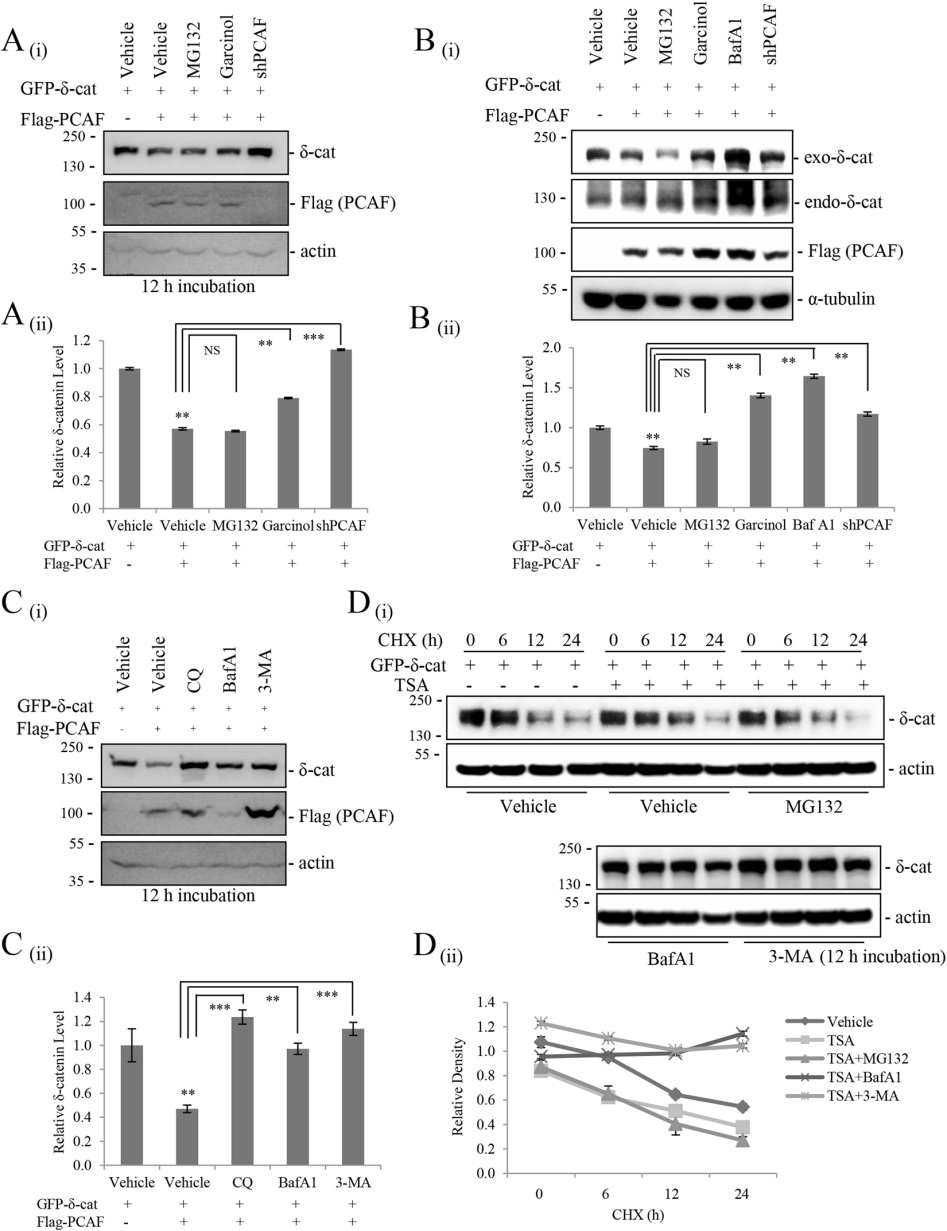
PCAF-mediated δ-catenin acetylation promotes autophagic degradation of δ-catenin. (**A**,**B**) The acetyltransferase activity of PCAF is required for the downregulation of δ-catenin. However, proteasome inhibition does not suppress the effect of PCAF on downregulating δ -catenin. HEK293Tcells transfected with GFP-δ-catenin and Flag-PCAF (**A**) and Rv/δ cells transfected with Flag-PCAF (**B**) were treated with the proteasome inhibitor MG132 (10 μM) or the histone acetyltransferase inhibitor Garcinol (5 µM) or Baf A1 (100 nM) or transfected with shPCAF and incubated for 12 h, and then cell lysates were subjected to immunoblotting. (**C**) Autophagy inhibitors attenuate PCAF-mediated δ-catenin degradation. HEK293T cells were transfected with the indicated plasmids. At 12 h post-transfection, cells were treated with the autophagy inhibitors chloroquine (CQ, 100 μM), bafilomycin A1 (BafA1, 100 nM), or 3-methyladenine (3-MA, 5 mM), and cell lysates were subjected to immunoblotting. (**D**) Autophagy inhibitors increase the stability of δ-catenin. HEK293T cells transfected with δ-catenin and treated with 0.2 µM TSA were treated with MG132 (10 μM) or Baf A1 (100 nM) or 3-MA (5 mM) and incubated for 12 h, and then treated with cycloheximide (CHX, 20 ng/ml) for indicated time (h), and then cell lysates were subjected to immunoblotting. α-Tubulin or ß-actin was used as a loading control. Relative δ-catenin/actin ratios from at least three independent experiments are shown as a bar graph in each panel (ii). Values are presented as the mean ± SEM. **p < 0.01; ***p < 0.001; NS, no significant difference compared with the control group. “−”, Mock transfection or vehicle treatment.

To determine whether the acetyltransferase activity of PCAF is necessary for the downregulation of δ-catenin and explore the underlying mechanisms, the effects of several inhibitors were assessed. The HAT inhibitor garcinol restored the downregulation of exogenous and endogenous δ-catenin to some extent (Fig. 2A,B), which together with the results of Fig. 1, suggested that PCAF-mediated downregulation of δ-catenin is dependent on the acetylation status of δ-catenin. Treatment with the proteasome inhibitor MG132 did not restore the PCAF-mediated downregulation of δ-catenin, suggesting that the ubiquitin/proteasome pathway was not involved (Figs 2A,B and S2A). However, treatment with the autophagy inhibitor bafilomycin A1 (BafA1), which is a vacuolar-type H (+)−ATPase inhibitor that prevents the maturation of autophagic vacuoles by inhibiting the fusion between autophagosomes and lysosomes, markedly increased the levels of PCAF-downregulated exogenous and endogenous δ-catenin (Fig. 2B,C). In addition, treatment with chloroquine (CQ), a lysosomal lumen alkalizer that inhibits autophagy; 3-methyladenine (3-MA), a class III PI3K inhibitor that blocks the early steps of autophagosome formation; or E-64, an autophagy inhibitor that suppresses lysosomal proteases^26^, significantly restored PCAF-mediated δ-catenin downregulation, suggesting that PCAF-mediated downregulation of δ-catenin is dependent on the autophagy activity (Figs 2C and S1B). To determine whether the treatment of autophagy inhibitor enhances the stability of δ-catenin, the cycloheximide chase assay were performed. TSA treatment decreased δ-catenin level and accelerated its turnover, but co-treatment with MG132 did not restore the level and turnover (Fig. 2D). However, co-treatment of TSA with BafA1 or 3-MA greatly increased the stability of δ-catenin, and, therefore, these will delay the turnover of δ-catenin (Fig. 2D). Taken together, these results indicated that PCAF-mediated δ-catenin acetylation promote the degradation of δ-catenin via the autophagy pathway.

### PCAF promotes δ-catenin degradation through the Atg5/LC3 pathway

**Figure 3:**
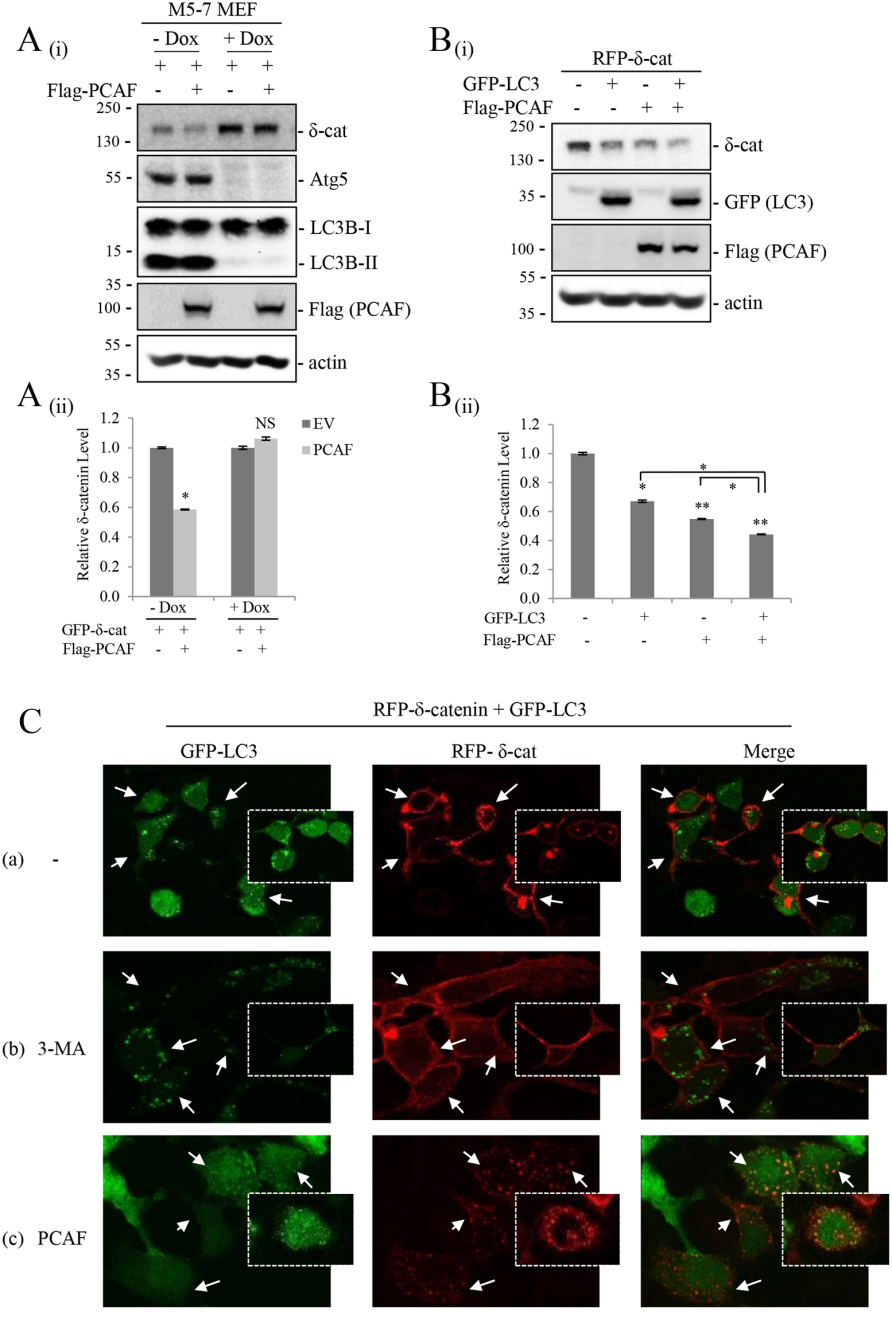
The Atg5/12-LC3 pathway is indispensable for PCAF-mediated δ-catenin degradation. (**A**) Atg5 is necessary for PCAF-mediated δ-catenin degradation. Tet-off Atg5−/− mouse embryonic fibroblasts m5-7 were cultured in the presence or absence of 10 ng/ml doxycycline (Dox) for 4 days. Then, cells were transfected as indicated for 24 h, and cell lysates were subjected to immunoblotting. (**B**) Overexpression of LC3 further decreases δ-catenin levels. HEK293T cells were transfected as indicated, and cell lysates were subjected to immunoblotting. Actin was used as a loading control. Relative values of δ-catenin/actin ratios from at least three independent experiments are shown as a bar graph in each panel (ii). Values are presented as the mean ± SEM. *p < 0.05; **p < 0.01; NS, no significant difference compared with the control group. (**C**) The cellular localization of δ-catenin depends on autophagic activity and PCAF. HEK293T cells were transiently transfected with a plasmid encoding GFP-LC3 and RFP-δ-catenin for 12 h, and incubated in the absence (a) or presence of 3-MA (b) or transfected with flag-PCAF (c) for 24 h. (a) Arrows indicate δ-catenin located at the plasma membrane or cell-cell contact region. (b) Arrows indicate cytoplasmic δ-catenin accumulation. 3-MA treatment dispersed LC3 signals and no colocalization with δ-catenin was observed. (c) Arrows indicated δ-catenin colocalization with LC3. PCAF overexpression decreased the accumulation of plasma membrane or cell-cell contact region and increased the colocalization with LC3. “−”, Mock transfection and/or vehicle treatment.

The involvement of autophagy in the degradation of acetylated δ-catenin was investigated using the Tet-off Atg5−/− MEF cell line m5-7^27^. In m5-7 MEFs, Atg5 expression and autophagic activity are completely lost in the presence of Dox and restored by Dox removal from the culture medium. As shown in Fig. 3A, the downregulation of δ-catenin by PCAF was evident in m5-7 MEFs in the absence of Dox. However, cells cultured in the presence of Dox showed δ-catenin accumulation (lane 1 vs. lane 3) and suppression of the PCAF effect (lane 3 vs. lane 4) (Fig. 3A). These results demonstrated that autophagic activity is necessary for the degradation of acetylated δ-catenin. To determine whether intracellular autophagic activity was a limiting factor in the degradation of δ-catenin, the effect of LC3 overexpression was assessed. As LC3, a mammalian homolog of yeast Atg8, is essential for autophagosome formation, overexpression of LC3 should promote the degradation of δ-catenin. The results showed that overexpression of LC3 alone or together with PCAF further decreased δ-catenin levels (Fig. 3B). Fluorescence microscopy showed that δ-catenin localized mostly to the plasma membrane and cell-cell contacts (Fig. 3C, arrow in panel a). Treatment of 3-MA, which blocks autophagosome formation, led to increased plasma membranous and cytoplasmic δ-catenin, but no evident colocalization of δ-catenin with LC3 was observed probably because LC3 cannot form functional autophagosome under 3-MA treatment (Fig. 3C, arrow in panel b). PCAF overexpression decreased plasma membranous δ-catenin and increased colocalization of δ-catenin with LC3 at cytoplasm (Fig. 3C, arrow in panel c). Taken together, these results demonstrated that the Atg5/LC3 machinery mediates the degradation of acetylated δ-catenin.

### Multiple N-terminal Lys residues of δ-catenin are responsible for PCAF-mediated δ-catenin downregulation

**Figure 4:**
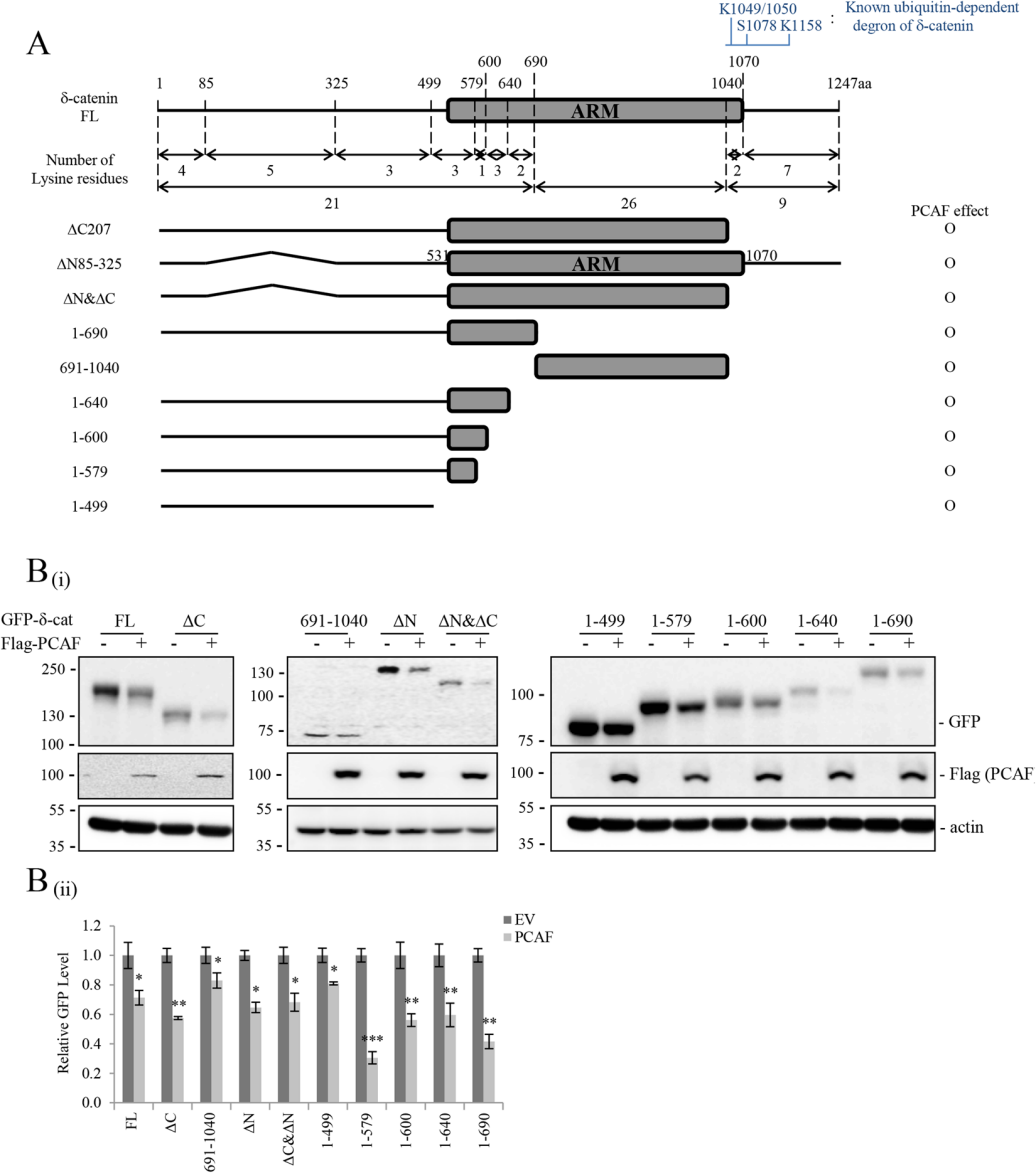
Multiple domains of δ-catenin are responsible for PCAF-mediated δ-catenin downregulation. (**A**) Schematic diagram of different constructs of full length GFP-δ-catenin and its deletion mutants (ΔC207, ΔN85–325, ΔN&ΔC, 1–690, 691–1040, 1–640, 1–600, 1–579, and 1–499) and the distribution of Lys residues on these deletion mutants. Lys residues (Lys1049, Lys1050, and Lys1158) form the ubiquitin-dependent degron of δ-catenin. (**B**) Both 1–690 and 691–1040 deletion mutants of δ-catenin were downregulated by PCAF. HEK293T cells were transfected with different combinations of the indicated plasmids, and cell lysates were subjected to immunoblotting. Actin was used as a loading control. Relative values of δ-catenin/actin ratios from at least three independent experiments are shown as a bar graph in each panel (ii). Values are presented as the mean ± SEM. *p < 0.05; **p < 0.01; ***p < 0.001; NS, no significant difference compared with the control group. “−”, Mock transfection.

**Figure 5:**
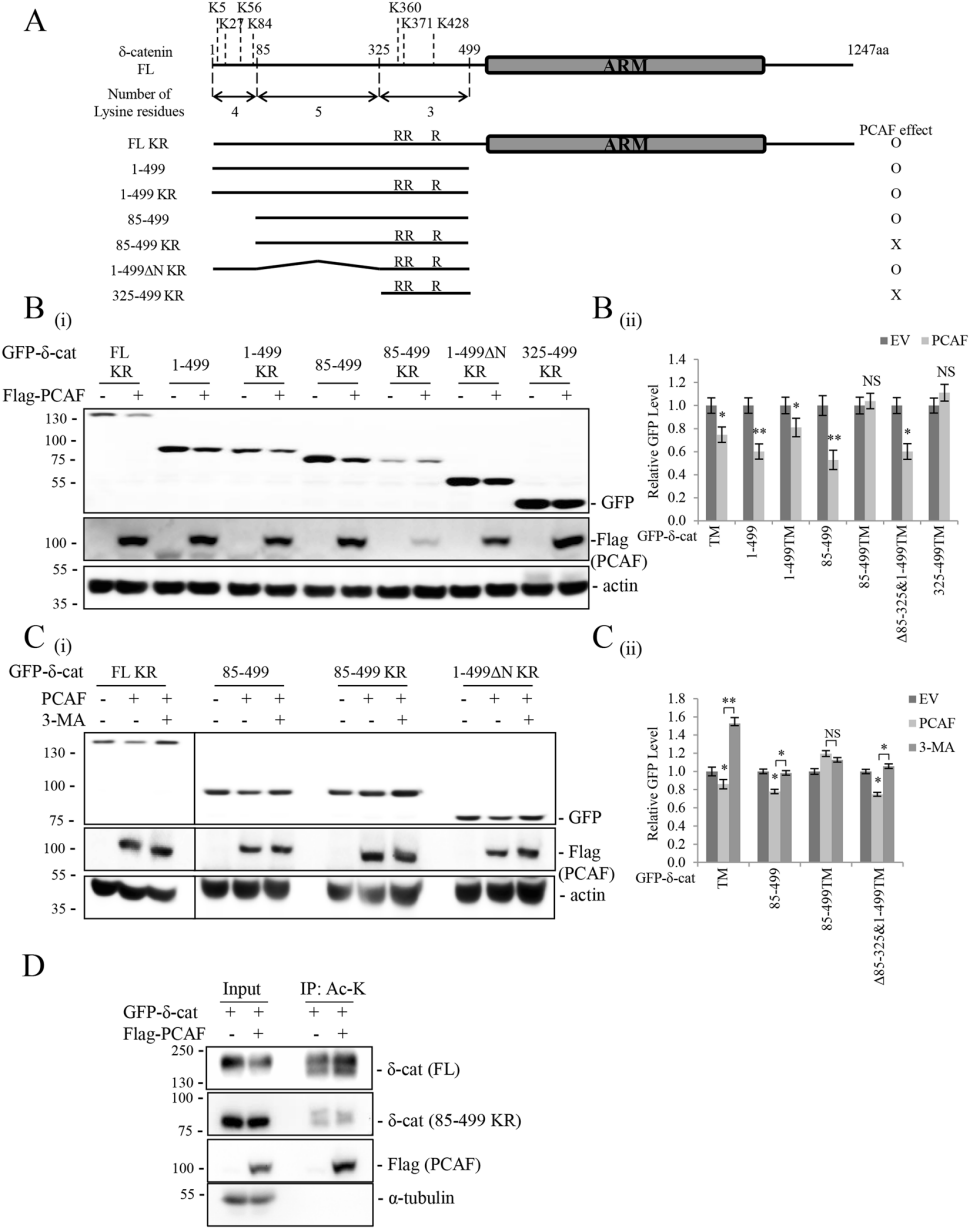
Multiple lysine residues in the N-terminus are responsible for PCAF-mediated δ-catenin downregulation. (**A**) Schematic representation of the triple arginine mutation at Lys360, Lys371, and Lys428 (FL KR), and the deletion/arginine mutation constructs of δ-catenin 1–499, 1–499 KR, 85–499, 85–499 KR, 1–499∆N KR, and 325–499 KR. (**B**) Deletion mutants 85–499 KR and 325–499 KR of δ-catenin were not affected by PCAF. HEK293T cells were transfected with the indicated plasmids expressing δ-catenin constructs, and cell lysates were subjected to immunoblotting with anti-GFP and anti-Flag antibody. (**C**) 3-MA restored the downregulated FL KR, 85–499, and 1–499∆N KR mutants of δ-catenin except the 85–499 KR mutation. HEK293T cells were transfected with the indicated plasmids expressing δ-catenin constructs and incubated with 3-MA (1 mM) for 24 h, and cell lysates were subjected to immunoblotting with anti-GFP and anti-Flag antibodies. (**D**) PCAF did not acetylate δ-catenin 85–499 KR mutation. HEK293T cells were transfected with full length GFP-δ-catenin or 85–499 KR mutant together with or without Flag-PCAF, and each cell lysates were subjected to immunoprecipitation with anti-acetylated-lysine, followed by immunoblotting of precipitated proteins. α-Tubulin or ß-actin was used as a loading control. Relative values of δ-catenin/actin ratios from at least three independent experiments are shown as a bar graph in each panel (ii). Values are presented as the mean ± SEM. *p < 0.05; **p < 0.01; NS, no significant difference compared with the control group. “−”, Mock transfection or vehicle treatment.

Next, we identified the Lys residue(s) in δ-catenin that are acetylated by PCAF. Several deletion mutants of δ-catenin including ΔN85–325, ΔC207, ΔN&ΔC, 1–690, and 691–1040 were generated in previous studies^4,28^, and the distribution of Lys residues in these deletion mutants is shown in Fig. 4A. Briefly, 1–690 of δ-catenin has 21 Lys residues; 691–1040 of δ-catenin has 26 Lys residues; and 1040–1247 of δ-catenin has nine Lys residues. Deletion of the N-terminal amino acids 85–325 (ΔN85–325) removes five Lys residues; deletion of C-terminal 207 (ΔC207) removes nine Lys residues, among which at least three Lys residues (Lys1049, Lys1050, and Lys1158) are major β-TrCP-1-mediated ubiquitination sites of δ-catenin after phosphorylation by glycogen synthase kinase-3β (GSK-3β) at Thr1078^4^ (Fig. 4A). In the present study, deletion of 1040–1247 or 85–325 of δ-catenin had no effect on PCAF-mediated δ-catenin downregulation, suggesting that the ubiquitin-dependent degron of δ-catenin at the C-terminus and the N-terminal five Lys residues in amino acids 85–325 are not involved in the acetylation-mediated degradation of δ-catenin by autophagy (Fig. 4B). However, the fact that the 1–690 and 691–1040 deletion mutants of δ-catenin are affected by PCAF suggests the involvement of more than one domain in PCAF-mediated acetylation and degradation. Since Lys residues are distributed over a wider range at the N-terminal 1–690 amino acids of δ-catenin, we constructed serial deletion mutants of δ-catenin harboring different numbers of Lys residues and examined the effect of PCAF. As shown in Fig. 4B, even the 1–499 deletion mutant was downregulated by PCAF, suggesting that four Lys residues in the 1–85 region and/or three Lys residues in the 325–499 region of δ-catenin are involved in PCAF-mediated acetylation and degradation. To identify the specific Lys residues acetylated, we generated additional deletion or arginine substitution mutants of δ-catenin harboring a smaller number of Lys residues and examined the effect of PCAF (Fig. 5A). Deletion of 1–85 of δ-catenin together with triple arginine substitutions at Lys360, Lys371, and Lys 428 (85–499 KR and 325–499 KR) abolished the effect of PCAF on downregulating δ-catenin (Fig. 5B). The presence of Lys residues among amino acids 85–325 (Lys111, Lys161, Lys276, Lys298, and Lys306) in the deletion mutants (85–499 KR) had no effect on PCAF-mediated downregulation. Although detection of acetylation on the indicated Lys residues by LC/MS/MS analysis or arginine substitution of each Lys residue was not performed, these results suggest that N-terminal Lys5/27/56/84/360/371/428 are responsible for PCAF-mediated acetylation and degradation of δ-catenin. Supporting these results, 3-MA treatment restored the downregulated δ-catenin mutants to normal levels except in the 85–499 KR mutant δ-catenin (Fig. 5C). Also, the level of 85–499 KR mutatnt acetylation was marginal and irrespondable to PCAF co-transfection (Fig. 5D).

### PCAF decreases the growth and motility of prostate cancer cells by suppressing oncogenic δ-catenin activity via δ-catenin downregulation

**Figure 6:**
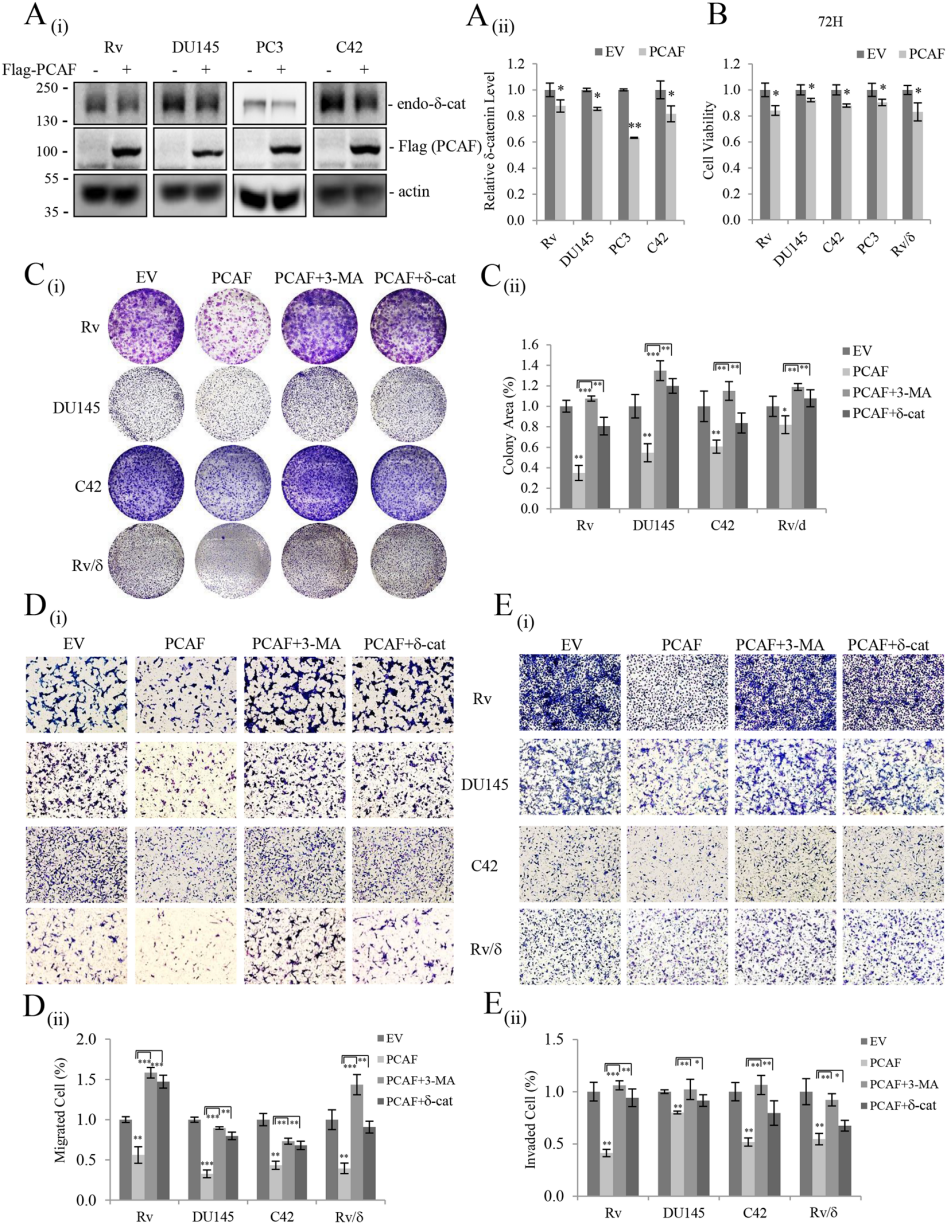
PCAF-mediated δ-catenin downregulation suppresses prostate cancer cell growth and motility. (**A**) PCAF reduced endogenous δ-catenin levels in Rv, DU145, PC3, and C42 prostate cancer cell lines. Four prostate cancer cell lines were transfected with empty vector or Flag-PCAF and subjected to immunoblotting with anti-δ-catenin or anti-Flag antibody. (**B**–**C**) PCAF suppressed the viability and growth of Rv, DU145, PC3, C42, and Rv/δ cells, and 3-MA treatment or δ-catenin overexpression restored the decreased growth of PCAF-overexpressing prostate cancer cells. Cell viability (**B**) and cell growth (**C**) were determined by the MTT assay and clonogenic assay, respectively, in empty vector or Flag-PCAF transfected cells treated or transfected with vehicle or 3-MA or δ-catenin. (**D**–**E**) PCAF reduced the migration and invasion abilities of Rv, DU145, C42, and Rv/δ cells while 3-MA treatment or δ-catenin overexpression restored the decreased migration and invasion of PCAF-overexpressing prostate cancer cells. Cells were transfected with Flag-PCAF together with or withiout δ-catenin or empty vector, and migration and invasion assays were performed in the presence of vehicle or 3-MA with Transwell chambers coated without (**D**) or with (**E**) gelatin, respectively. Quantitative analysis of δ-catenin levels, cell viability, colony area, and migrated and invaded cell numbers from at least three independent experiments are shown as a bar graph in each panel (ii). Values are presented as the mean ± SEM. *p < 0.05; **p < 0.01; ***p < 0.001; NS, no significant difference compared with the control group. “−”, Mock transfection.

To determine whether PCAF affects the expression and thus the oncogenic activity of endogenous δ-catenin in prostate cancer cells, several prostate cancer cell lines with different metastatic potential, including CWR22Rv-1 (Rv), DU145, PC3, and C42, were analyzed. As shown in Fig. 6A, PCAF overexpression downregulated endogenous δ-catenin in all the tested cells albeit to different extents. Consequently, the viability and growth of PCAF-overexpressing prostate cancer cells were lower than those of empty vector transfected cells (Fig. 6B,C). The migration and invasion abilities were also significantly lower in PCAF-overexpressing than in control prostate cancer cells (Fig. 6D,E). Moreover, 3-MA treatment or δ-catenin overexpression restored the decreased growth, migration, and invasion of PCAF-overexpressing prostate cancer cells (Fig. 6C–E). Taken together, these results demonstrated that PCAF downregulates δ-catenin and thus decreases the tumorigenicity and motility of prostate cancer cells.

**Figure 7:**
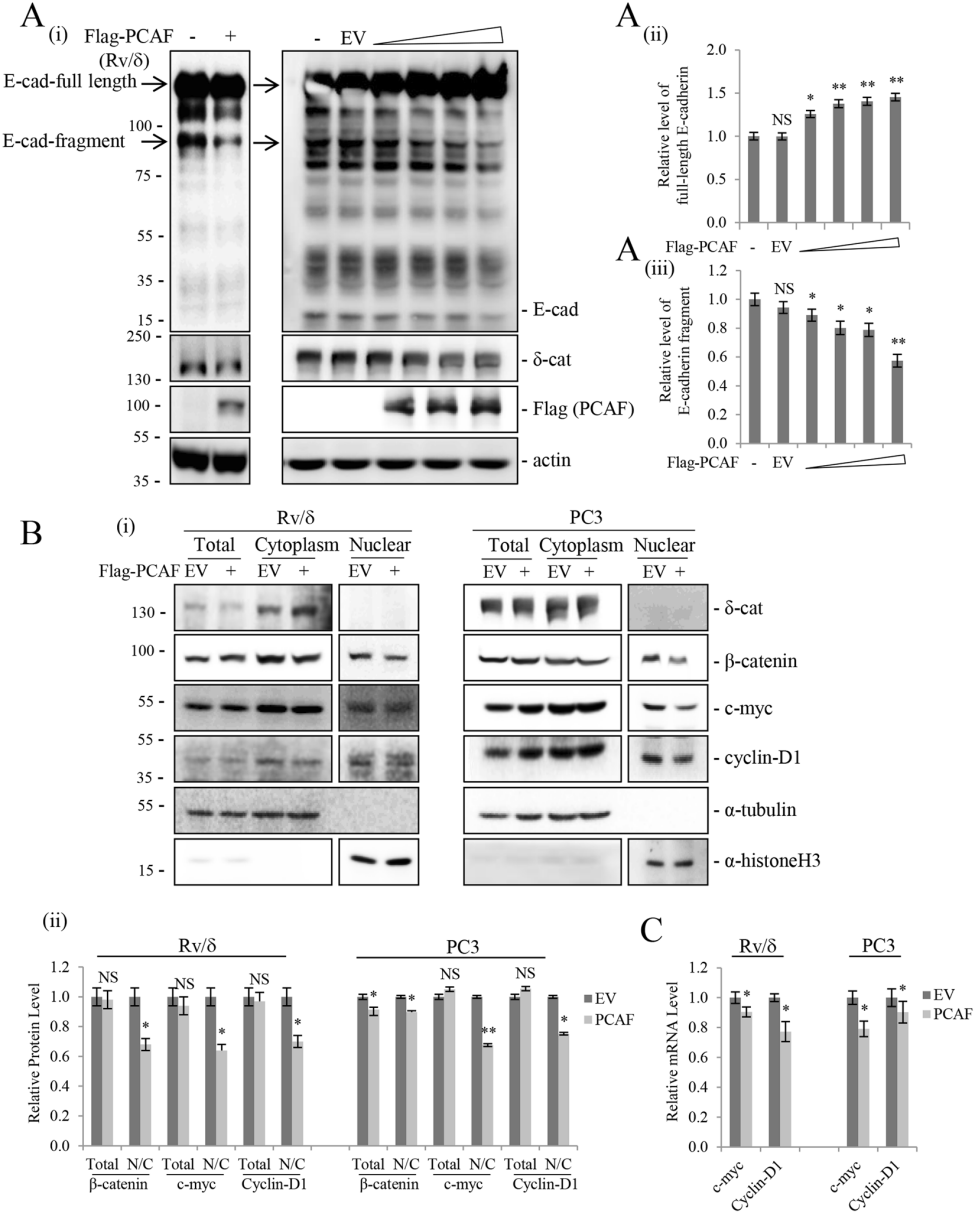
PCAF inhibits E-cadherin processing and inactivates β-catenin-mediated signaling. (**A**) PCAF-mediated δ-catenin downregulation reduced E-cadherin processing. PCAF decreased E-cadherin fragment levels in a dose-dependent manner, whereas total E-cadherin protein levels increased. E-cadherin processing was measured in Rv/δ cells transfected with increasing doses of Flag-PCAF. (**B**) PCAF suppressed subcellular β-catenin distribution and its downstream effectors, c-myc and cyclin-D1. Nuclear and cytoplasmic fractionation of β-catenin, c-myc, and cyclin-D1 was performed in Rv/δ and PC3 cells transfected with Flag-PCAF. Moderate changes in total β-catenin, c-myc, and cyclin-D1 levels were also observed. α-Tubulin was used as a cytoplasmic marker, and α-histone H3 was used as a nuclear marker. Relative values from at least three independent experiments are shown as a bar graph in each panel (ii/iii). (**C**) PCAF decreased β-catenin target gene levels. The mRNA levels of c-myc and cyclin-D1 were analyzed by qRT-PCR in Rv/δ and PC3 cells transfected with Flag-PCAF. Values are presented as the mean ± SEM. *p < 0.05; **p < 0.01; NS, no significant difference compared with the control group. “−”, No transfection.

In our previous study, we showed that the oncogenic activity of δ-catenin in prostate cancer is mediated by promoting E-cadherin processing and the subsequent transactivation of β-catenin-mediated signaling^5^. In the present study, PCAF overexpression dose-dependently decreased E-cadherin processing in Rv/δ cells (Fig. 7A). Namely, PCAF overexpression in Rv/δ cells increased the level of FL E-cadherin (Fig. 7A ii) and decreased the levels of E-cadherin fragments (Fig. 7A iii). Furthermore, PCAF-overexpressing Rv/δ and PC3 cells showed decreased nuclear distribution of β-catenin and decreased nuclear distribution and expression of downstream target genes such as c-myc and cyclin-D1 compared with those in empty vector transfected cells (Fig. 7B). Taken together, these results suggest that PCAF-mediated δ-catenin downregulation decreases cell growth and motility, thereby suppressing the oncogenic activity of δ-catenin in prostate cancer cells.

## Discussion

Several studies reported the upregulation of δ-catenin in certain carcinomas, which suggests that the regulation of δ-catenin stability is a key event in cancer progression. In the present study, we investigated the molecular mechanisms underlying the acetylation and autophagic degradation of δ-catenin. The major findings of the study were as follows: (1) PCAF and HDACs regulate the acetylation status of δ-catenin; (2) acetylated δ-catenin is degraded through the Atg5/LC3-mediated autophagosomal pathway; (3) multiple Lys residues in the N-terminus of δ-catenin are involved in PCAF-mediated δ-catenin acetylation; (4) PCAF-mediated δ-catenin degradation decreases the growth and motility of prostate cancer cells; and (5) PCAF-mediated δ-catenin degradation inhibits E-cadherin processing and inactivates β-catenin-mediated signaling. The present results indicate that acetylation-dependent autophagic δ-catenin degradation by PCAF reduces oncogenic δ-catenin signaling, as indicated by E-cadherin processing and β-catenin-mediated signals, thereby inhibiting the progression of prostate cancer (Fig. S3).

δ-Catenin shares many characteristics with β-catenin, and the two proteins have common binding partners^29,30^. Studies show that PCAF acetylates and stabilizes β-catenin, upregulating the expression of Wnt-responsive genes^21,31^. The present study is the first to provide evidence that PCAF acetylates δ-catenin leading to its downregulation, whereas HDACs deacetylate and upregulate δ-catenin. Acetylation of Lys residues protects them from ubiquitination, which stabilizes proteins in most cases^32^. There are few examples of acetylation resulting in protein destabilization. Acetylation destabilizes fatty acid synthase (FASN), which inhibits *de novo* lipogenesis and tumor cell growth, suggesting that targeting FASN acetylation could be an anticancer strategy^33^. ARD1-mediated acetylation destabilizes hypoxia-inducible factor 1 (HIF-1α)^34^. These studies illustrate the effect of acetylation on promoting ubiquitination-dependent proteasomal degradation of proteins, and suggest that acetylation-dependent destabilization plays an important role in tumorigenicity. The results of the present study support that acetylation can mediate protein destabilization. However, in contrast to previous reports, acetylation of δ-catenin promoted autophagic degradation and decreased oncogenic δ-catenin signaling.

Studies indicate that the degradation of δ-catenin is mediated by the ubiquitin-proteasome pathway^4^. Phosphorylation of δ-catenin by GSK-3β at Thr1078 enables β-TrCP-mediated ubiquitination of δ-catenin, targeting δ-catenin for degradation by the ubiquitin-proteasome pathway. In a previous study, the degradation of δ-catenin by the lysosomal pathway was suggested by the increased level of δ-catenin in response to CQ treatment^4^; however, the exact mechanisms underlying lysosomal δ-catenin degradation remained elusive. In the present study, treatment with the proteasome inhibitor MG132 did not restore PCAF-mediated δ-catenin downregulation. However, treatment with autophagy inhibitors with various mechanisms of action, such as blocking the early step of autophagosome formation (3-MA), the maturation (BafA1), and the autophagic process (CQ and E-64)^26^, restored PCAF-mediated δ-catenin downregulation, suggesting that δ-catenin acetylation is required for the initial step of the autophagosomal degradation process. Consistently, conditional knockdown of Atg5 suppressed PCAF-mediated δ-catenin downregulation, and this effect was reversed when Atg5 expression was restored. Overexpression of LC3 further decreased δ-catenin levels, and PCAF increased the colocalization of LC3 with δ-catenin. These results indicate that acetylated δ-catenin by PCAF can undergo degradation via the autophagosomal pathway, which is related to the expression of the autophagosomal marker Atg5/LC3. Autophagosomal targeting of non-histone proteins by acetylation was reported previously^35^. Acetylation of mutant Huntingtin (Htt) at Lys444 facilitates trafficking of mutant Htt into autophagosomes, increasing the clearance of Htt by autophagy. The identification of the acetylation site on δ-catenin is important. However, despite a series of experiments with deletion and arginine substitution mutants, we were only able to show that multiple Lys residues in the N-terminus and ARM repeat domain of δ-catenin are responsible for PCAF-mediated δ-catenin downregulation.

δ-Catenin is overexpressed in several carcinomas, and its ability to interact with E-cadherin underlies the oncogenic activity of δ-catenin in epithelial-derived carcinoma cells^36,37^. Our previous study demonstrated that prostate cancer cell lines overexpressing δ-catenin display noticeable multi-layer growth, E-cadherin processing, and nuclear β-catenin localization, with the subsequent activation of β-catenin downstream target genes^5^. PCAF-mediated δ-catenin acetylation/destabilization is involved in prostate cancer progression by suppressing E-cadherin processing and β-catenin-mediated oncogenic signals, thereby inhibiting prostate cancer cell motility and tumorigenesis. The present investigation of the mechanism of δ-catenin acetylation provides a new perspective on the process of δ-catenin degradation and improves our understanding of the effects of δ-catenin on E-cadherin or β-catenin, which is implicated in cancer progression.

The results of the present study indicate that PCAF inhibits the progression of carcinoma by promoting the acetylation-mediated degradation of δ-catenin and the suppression of its oncogenic signaling. However, PCAF affects non-histone proteins as an acetyltransferase involved in cancer, and acts as both oncogene and tumor suppressor. PCAF can function as a cofactor for several proto-oncogenes. PCAF possesses intrinsic ubiquitination activity that is critical for the control of Hdm2 expression levels, and thus p53 function^38^. These findings highlight the complexity of nucleosome modification associated with proto-oncogene induction, and identify HATs as key factors in this process. PCAF is also involved in virus-induced tumorigenesis. The oncogenic effect of human papillomavirus E7 (zinc-finger mutation) is dependent on PCAF and its deregulated HAT function in tumor development^39^. However, PCAF also functions as a tumor-suppressor gene, and its loss of function causes tumorigenesis. PCAF can induce cell apoptosis by modulating the GLI1/BCL-2/BAX axis or by acetylating histone H4 and inactivating AKT signaling, which in turn suppresses hepatocellular carcinoma progression^40,41^. PCAF-mediated acetylation of the transcriptional factor HOXB9 suppresses lung adenocarcinoma progression^42^. Studies suggest that PCAF modulates the activity or stability of several oncogenes or tumor suppressors through the acetylation of histones or transcription factors, consequently impacting cancer progression. The present study demonstrated that PCAF suppresses δ-catenin-mediated oncogenic signals by promoting δ-catenin degradation through the autophagy pathway, thereby suppressing prostate cancer cell growth and motility.

## Methods

### Plasmids

The construction of N-terminal GFP-tagged δ-catenin full length (FL), ∆N85–325, ∆C207, ∆N85–325&∆C207, 1–690, and 691–1040 in pEGFP-C1 and RFP-tagged δ-catenin was previously described^4,43^. The deletion mutants of δ-catenin (1–499, 1–579, 1–600, and 1–640) and the point mutants of δ-catenin, in which lysine (Lys) residue(s) are substituted for arginine, were generated using a PCR-based exchange site-directed mutagenesis kit (Enzynomic, Daejeon, Korea). All constructs were confirmed by sequencing. The p300, PCAF, and monocytic leukemic zinc finger (MOZ) expression plasmids, and shPCAF, were kindly provided by Prof. Nacksung Kim (Chonnam National University Medical School, Gwangju, Korea), and histone deacetylase (HDAC) 1–9 expression plasmids were kindly provided by Prof. Hyun Kook (Chonnam National University Medical School, Gwangju, Korea). GFP-tagged LC3 expression plasmids were kindly provided by Prof. Kwang Youl Lee (Chonnam National University, Gwangju, Korea).

### Antibodies

The following antibodies were used in the present study: anti-δ-catenin (#611537, BD Bioscience, San Jose, CA, USA), anti-GFP (#G1544, Santa Cruz Biotechnology, Santa Cruz, CA, USA), anti-tubulin (#T9026, Sigma-Aldrich, St Louis, MO, USA), anti-lamin B (SC-6216, Santa Cruz Biotechnology), anti-ß-actin (Santa Cruz Biotechnology), anti-flag (Sigma-Aldrich), anti-PCAF (SC-13124, Santa Cruz Biotechnology), anti-E-cadherin (BD Bioscience), anti-acetylated-Lys (Cell Signaling, Beverly, MA, USA), anti-p120-δ-catenin (BD Bioscience), anti-autophagy-related protein 5 (Atg5) (Cell Signaling), anti-myc (Santa Cruz Biotechnology), and anti-cyclin-D1 (Calbiochem, San Diego, CA, USA).

### Cell culture and transfection

Human embryonic kidney HEK293T cells were cultured in DMEM (Gen Depot, Barker, TX, USA) supplemented with 10% fetal bovine serum (Gen Depot) and 1% penicillin-streptomycin solution. The human prostate cancer cells CWR22Rv-1 (RV1), DU145, C42, and PC3 were cultured in RPMI1640 culture medium (Gen Depot) supplemented with 10% fetal bovine serum (Gen Depot) and 1% penicillin-streptomycin solution. Tet-Off Atg5 conditional knockout m5–7 mouse embryonic fibroblasts (MEFs) were kindly provided by Dr. N. Mizushima (Tokyo University, Japan)^27^ and cultured in DMEM containing 10% (v/v) FBS with or without 10 ng/ml doxycycline (Dox; Sigma-Aldrich). Cells were cultured in 5% CO_2_ in a humidified atmosphere at 37 °C. HEK293T cells were transfected with X-tremeGENE 9 DNA Transfection Reagent (Roche, Sandhofer, Mannheim, Germany), and M6A and PC3 cells were transfected with Lipofectamine Plus reagent (Invitrogen, Carlsbad, CA, USA) according to the manufacturer’s instructions. For protein stability assay, cycloheximide (Sigma-Aldrich) dissolved in DMSO was used.

### Immunoblotting and immunoprecipitation

Immunoblotting was performed as previously described^4^. Immunoprecipitation was performed using lysates from transfected cells that were incubated with primary antibodies overnight at 4 °C and pulled down with Protein A/G Sepharose (Thermoscientific, Rockford, IL, USA) for 3 h. The immunoprecipitated proteins were washed twice with the same buffer, and bound proteins were resolved by SDS-PAGE followed by immunoblotting.

### Cell viability assays

The MTT [3-(4, 5-dimethylthiazol-2-yl)-2, 5-diphenyltetrazolium bromide, Roche] assay was used to measure cell viability. In total, 2 × 10^3^ human prostate cancer (RV1, DU145, C42, and PC3) cells were plated at least in triplicate in 96-well plates and transfected with PCAF-expressing plasmid or empty vector. After incubating 72 h, 0.5 mg/ml MTT was added to each well and incubated for an additional 4 h. Then, the blue MTT formazan precipitate was dissolved in 100 μl of DMSO. The absorbance at 540 nm was measured on a multi-well plate reader.

### Clonogenic assay

The clonogenic assay was performed as previously described^44^. After transfection, the cells were seeded in 6-well plates at a density of 3000 cells/well and cultured for 9–14 days until visible colonies appeared. Then, the cells were stained with crystal violet, and colonies containing more than 50 cells were counted. The pixel intensity of the colony area was measured by the IMT iSolution software (IMT i-Solution Inc., Northampton, NJ, USA) in randomly selected microscope fields in each plate. To measure the percent area of colonies, the pixel amount of each colony area was normalized to pixel × pixel square. Data represent the mean of three experiments.

### Transwell migration and invasion assays

Migration and invasion assays were performed in Transwell chambers (Corning, New York, USA) with 8 μm pore size polycarbonate membranes. For invasion assays, the upper chamber was coated with 1% gelatin. Cells transfected with flag-PCAF or empty vector were plated at a density of 2–2.5 × 10^5^ cells/well in RPMI1640/DMEM containing 0.2% bovine serum albumin (BSA) in the upper compartment of the chamber. RPMI1640/DMEM containing 10 μg/ml fibronectin was then added to the lower chamber as a chemotactic agent. After 24 h of incubation, upper chamber cells were fixed with a Diff Quik kit (Sysmex, Kobe, Japan). The cells inside the chamber were removed from the membrane with a cotton swab, and the cells adhering to the underside of the membrane were stained and counted under a light microscope (five fields per chamber). Quantification of migrated and invaded cells was performed as previously described^45^. Each assay was repeated in three independent experiments. The results are expressed as the mean number of cells migrating per high-power field.

### Cell fractionation

Cell fractionation was performed as previously described^46^. Extracts of cytoplasm and nuclear were separated using the NE-PER nuclear and cytoplasmic extraction kit (PIERCE Biotechnology, Waltham, MA, USA). Briefly, harvested cells were resuspended in ice-cold CER I, followed by ice-cold CER II. Then, lysates were homogenized and centrifuged at 16,000 g for 5 min at 4 °C, and the supernatant was collected as the cytoplasmic fraction. The remaining pelleted lysates were resuspended in ice-cold NER within 40 min and centrifuged at maximum speed, and the supernatant was collected as the nuclear fraction. All extracts were stored at −80 °C until use.

### qRT-PCR

Briefly, total RNA was isolated from human prostate cancer cells transfected with flag-PCAF or empty vector using RNAiso Plus (TaKaRa, Otsu, Shiga 520–2193, Japan) according to the manufacturer’s instructions. 1 μg of total RNA from each group of transfected cells was synthesized to cDNA using a M-MLV Reverse Transcriptase Kit (Invitrogen) and SYBR green (Enzynomics, Seoul, Korea). The qRT-PCR reaction and data analysis were performed using CFX (Bio-Rad, Hercules, CA, USA).

### Statistical analysis

Data are expressed as the mean ± SD. Results comparing three or more samples were analyzed using analysis of variance and Bonferroni’s tests; comparisons between two samples were analyzed using Student’s t-tests. Probabilities of p < 0.05 were considered statistically significant.

## Supplementary information

Dataset 1

## References

[1] Shibamoto S (1995). Association ofp120, a tyrosine kinase substrate, with E-cadherin/catenin complexes. The Journal of cell biology.

[2] Paffenholz R, Franke WW (1997). Identification and localization of a neurally expressed member of the plakoglobin/armadillo multigene family. Differentiation; research in biological diversity.

[3] Lu Q (2005). Increased expression of δ-catenin/neural plakophilin-related armadillo protein is associated with the down-regulation and redistribution of E-cadherin and p120 ctn in human prostate cancer. Human pathology.

[4] Shrestha H (2016). Investigation of the molecular mechanism of delta-catenin ubiquitination: Implication of beta-TrCP-1 as a potential E3 ligase. Biochimica et biophysica acta.

[5] Kim H (2012). delta-Catenin promotes E-cadherin processing and activates beta-catenin-mediated signaling: implications on human prostate cancer progression. Biochimica et biophysica acta.

[6] Israely I (2004). Deletion of the neuron-specific protein delta-catenin leads to severe cognitive and synaptic dysfunction. Current biology: CB.

[7] Arikkath J (2009). Delta-catenin regulates spine and synapse morphogenesis and function in hippocampal neurons during development. The Journal of neuroscience: the official journal of the Society for Neuroscience.

[8] Zeng Y (2009). δ-Catenin promotes prostate cancer cell growth and progression by altering cell cycle and survival gene profiles. Molecular cancer.

[9] Wang M, Dong Q, Zhang D, Wang Y (2011). Expression of delta-catenin is associated with progression of human astrocytoma. BMC cancer.

[10] Zhang D, Zhang JY, Wang E (2015). H. delta-catenin promotes the malignant phenotype in breast cancer. Tumour biology: the journal of the International Society for Oncodevelopmental Biology and Medicine.

[11] Zhang JY (2010). δ‐Catenin promotes malignant phenotype of non‐small cell lung cancer by non‐competitive binding to E‐cadherin with p120ctn in cytoplasm. The Journal of pathology.

[12] Fang Y, Li Z, Wang X, Zhang S (2012). Expression and biological role of delta-catenin in human ovarian cancer. Journal of cancer research and clinical oncology.

[13] Zhang JY (2014). The expression of delta-catenin in esophageal squamous cell carcinoma and its correlations with prognosis of patients. Human pathology.

[14] Zhang H (2014). Delta-catenin promotes the proliferation and invasion of colorectal cancer cells by binding to E-cadherin in a competitive manner with p120 catenin. Targeted oncology.

[15] He Y (2013). δ-catenin overexpression promotes angiogenic potential of CWR22Rv-1 prostate cancer cells via HIF-1α and VEGF. FEBS letters.

[16] Baarsma HA, Königshoff M, Gosens R (2013). The WNT signaling pathway from ligand secretion to gene transcription: molecular mechanisms and pharmacological targets. Pharmacology & therapeutics.

[17] Lockhart DJ, Winzeler EA (2000). Genomics, gene expression and DNA arrays. Nature.

[18] Stern B, Olsen LC, Tröße C, Ravneberg H, Pryme IF (2007). Improving mammalian cell factories: The selection of signal peptide has a major impact on recombinant protein synthesis and secretion in mammalian cells. Trends Cell Mol Biol.

[19] Hunter T (2007). The age of crosstalk: phosphorylation, ubiquitination, and beyond. Molecular cell.

[20] Lévy L (2004). Acetylation of β-catenin by p300 regulates β-catenin-Tcf4 interaction. Molecular and cellular biology.

[21] Ge X, Jin Q, Zhang F, Yan T, Zhai Q (2009). PCAF acetylates β-catenin and improves its stability. Molecular biology of the cell.

[22] van Loosdregt J (2010). Regulation of Treg functionality by acetylation-mediated Foxp3 protein stabilization. Blood.

[23] Spencer TE (1997). Steroid receptor coactivator-1 is a histone acetyltransferase. Nature.

[24] Love IM, Sekaric P, Shi D, Grossman SR, Androphy EJ (2012). The histone acetyltransferase PCAF regulates p21 transcription through stress-induced acetylation of histone H3. Cell Cycle.

[25] Liu L (1999). p53 sites acetylated *in vitro* by PCAF and p300 are acetylated *in vivo* in response to DNA damage. Molecular and cellular biology.

[26] Yang YP (2013). Application and interpretation of current autophagy inhibitors and activators. Acta pharmacologica Sinica.

[27] Hosokawa N, Hara Y, Mizushima N (2006). Generation of cell lines with tetracycline-regulated autophagy and a role for autophagy in controlling cell size. FEBS letters.

[28] Kim K (2002). Dendrite-like process formation and cytoskeletal remodeling regulated by delta-catenin expression. Experimental cell research.

[29] He Y, Ki H, Kim H, Kim K (2015). delta-Catenin interacts with LEF-1 and negatively regulates its transcriptional activity. Cell biology international.

[30] Kim H, Ki H, Park HS, Kim K (2005). Presenilin-1 D257A and D385A mutants fail to cleave Notch in their endoproteolyzed forms, but only presenilin-1 D385A mutant can restore its gamma-secretase activity with the compensatory overexpression of normal C-terminal fragment. The Journal of biological chemistry.

[31] Hoffman J, Lee M, Davis CR, Kuo CJ (2004). Wnts as essential growth factors for the adult small intestine and colon. Cell Cycle.

[32] Shaw BF (2008). Lysine acetylation can generate highly charged enzymes with increased resistance toward irreversible inactivation. Protein Science.

[33] Lin, H.-P. *et al*. Destabilization of fatty acid synthase by acetylation inhibits de novo lipogenesis and tumor cell growth. *Cancer research* (2016). 10.1158/0008-5472.CAN-16-1597 PMC5135623 27758890

[34] Jeong J-W (2002). Regulation and destabilization of HIF-1α by ARD1-mediated acetylation. Cell.

[35] Jeong H (2009). Acetylation targets mutant huntingtin to autophagosomes for degradation. Cell.

[36] Ireton RC (2002). A novel role for p120 catenin in E-cadherin function. The Journal of cell biology.

[37] Lu Q (2010). delta-Catenin dysregulation in cancer: interactions with E-cadherin and beyond. The Journal of pathology.

[38] Linares LK (2007). Intrinsic ubiquitination activity of PCAF controls the stability of the oncoprotein Hdm2. Nature cell biology.

[39] Avvakumov N, Torchia J, Mymryk JS (2003). Interaction of the HPV E7 proteins with the pCAF acetyltransferase. Oncogene.

[40] Zheng X (2013). Histone acetyltransferase PCAF up-regulated cell apoptosis in hepatocellular carcinoma via acetylating histone H4 and inactivating AKT signaling. Molecular cancer.

[41] Gai X (2015). Histone acetyltransferase PCAF accelerates apoptosis by repressing a GLI1/BCL2/BAX axis in hepatocellular carcinoma. Cell death & disease.

[42] Wan J (2016). PCAF-mediated acetylation of transcriptional factor HOXB9 suppresses lung adenocarcinoma progression by targeting oncogenic protein JMJD6. Nucleic acids research.

[43] Kim K (2002). Dendrite-like process formation and cytoskeletal remodeling regulated by δ-catenin expression. Experimental cell research.

[44] Wang J (2014). A quantitative chemical proteomics approach to profile the specific cellular targets of andrographolide, a promising anticancer agent that suppresses tumor metastasis. Molecular & cellular proteomics: MCP.

[45] Zhou R (2017). The lichen secondary metabolite atranorin suppresses lung cancer cell motility and tumorigenesis. Scientific reports.

[46] Park, S. Y. *et al*. Glycoprotein 90K Promotes E-Cadherin Degradation in a Cell Density-Dependent Manner via Dissociation of E-Cadherin-p120-Catenin Complex. *International journal of molecular sciences* **18**, 10.3390/ijms18122601 (2017). 10.3390/ijms18122601 PMC5751204 29207493

