# Supplementary material for: p300/CBP-associated factor promotes autophagic degradation of δ-catenin through acetylation and decreases prostate cancer tumorigenicity
Source: Sci Rep. 2019 Mar 4;9:3351. doi: 10.1038/s41598-019-40238-w (PMC6399259; doi:10.1038/s41598-019-40238-w)
Supplement: Supplementary file 1 — Dataset 1 [file 41598_2019_40238_MOESM1_ESM.pdf]

Supplementary Data for

p300/CBP-associated factor promotes autophagic degradation of δ-catenin through acetylation and decreases prostate cancer tumorigenicity

Short title: Autophagic degradation of acetylated δ-catenin

Rui Zhou[a], Yi Yang[a], So-Yeon Park[a], Young-Woo Seo[b], Sang-Chul Jung[c], Kyung Keun Kim[d], Kwonseop Kim[e], Hangun Kim[a,*]

*Correspondence. Email: hangunkim@sunchon.ac.kr (HK)

Supplementary Data

Supplementary Figure 1. HATs downregulate δ-catenin levels.

(A) HEK293T cells were transfected with plasmids expressing GFP-δ-catenin (0.5 μg) alone or together with 1 μg of HA-p300, flag-PCAF, HA-MOZ, or empty vector. Lysates were subjected to immunoblotting using the indicated antibodies. (B) HEK293T cells were transfected with plasmids expressing GFP-δ-catenin alone or together with HA-p300, HA-p53, or empty vector, and cell lysates were subjected to immunoprecipitation with acetyl-lysine antibody followed by immunoblotting with a mixture of GFP and HA antibodies. “-”, mock transfection.

Supplementary Figure 2. Lysosomal activity, not proteasomal activity, is responsible for PCAF-mediated δ-catenin degradation.

(A) Proteasome inhibitor does not deprive PCAF effect on downregulating δ-catenin. HEK293T cells transfected with indicated plasmids were treated with a short time-course of MG132 (10 μM), and cell lysates were subjected to immunoblotting. (B) The lysosomal inhibitor E64 attenuated PCAF-mediated δ-catenin degradation. After 12 h post-transfection with the indicated plasmids, cells were treated with different concentrations of the lysosomal inhibitor E-64 for 48 h. Actin was used as a loading control. Relative δ-catenin/actin ratios from at least three independent experiments are shown as a bar graph in each panel (ii). Values are presented as the mean ± SEM. $^{**}p<0.01$; $^{***}p<0.001$; NS, no significant difference compared with indicated group. “-”, mock transfection.

Supplementary Figure 3. Schematic showing PCAF acetylates δ-catenin through

Atg5/12/LC3-autophagosomal degradation pathway, and thereby suppressing E-cadherin processing and β-catenin localization, further inhibiting prostate cancer progression.

Supplementary Figure 4. Full-length blots shown in Figure 1.

Supplementary Figure 5. Full-length blots shown in Figure 2.

Supplementary Figure 6. Full-length blots shown in Figure 3.

Supplementary Figure 7. Full-length blots shown in Figure 4.

Supplementary Figure 8. Full-length blots shown in Figure 5.

Supplementary Figure 9. Full-length blots shown in Figure 6.

Supplementary Figure 10. Full-length blots shown in Figure 7.

Supplementary Figure 11. Full-length blots shown in Supplementary Figure 1.

Supplementary Figure 12. Full-length blots shown in Supplementary Figure 2.

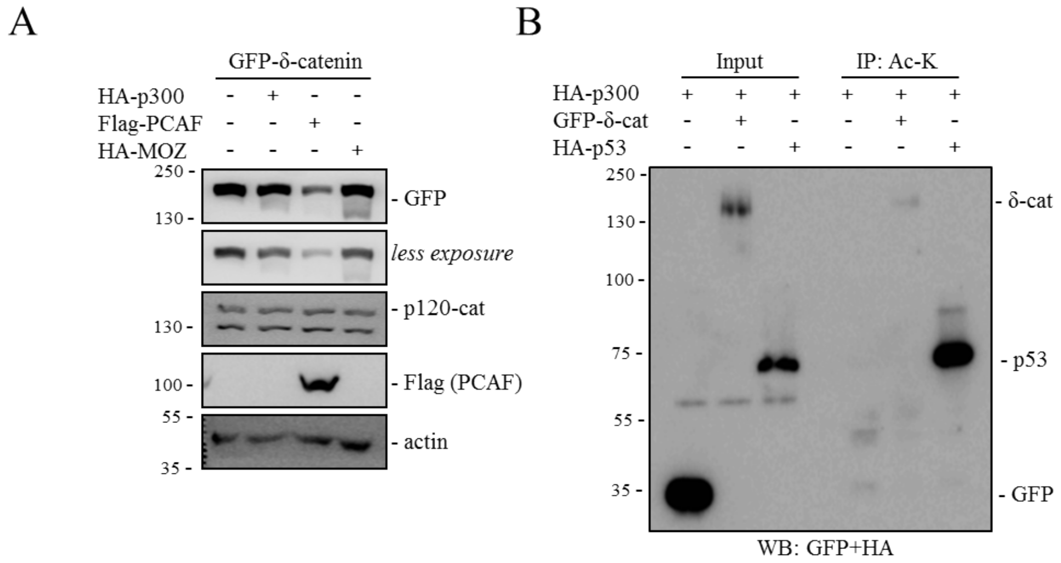

Supplementary Figure 1

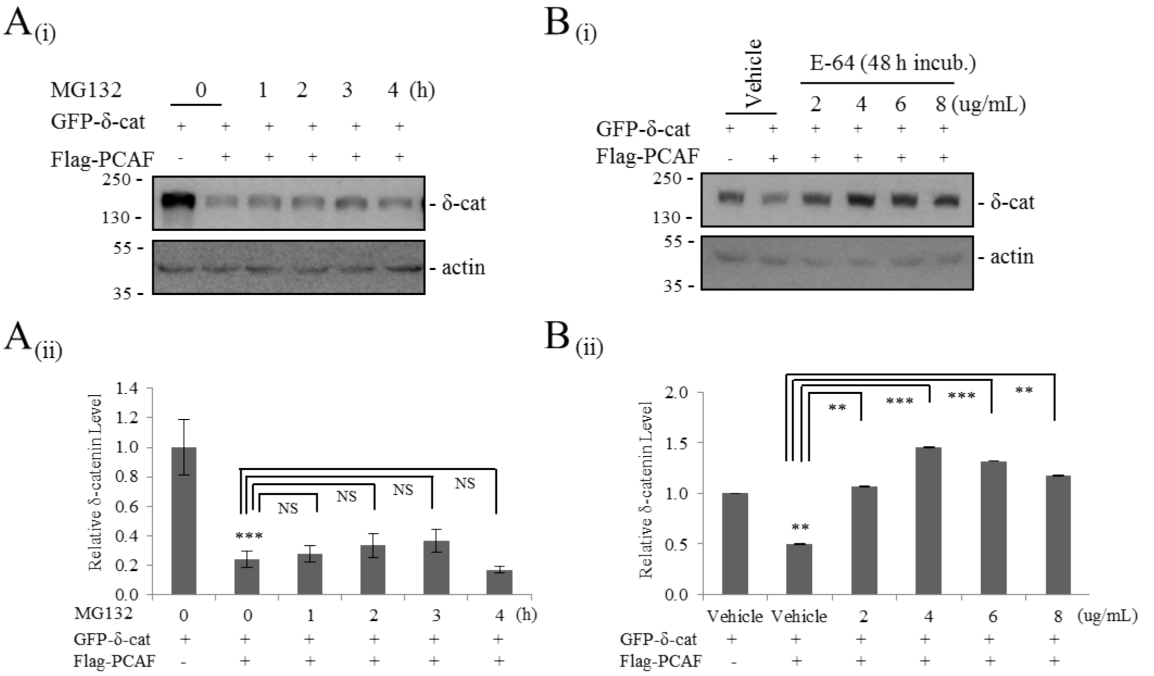

Supplementary Figure 2

A

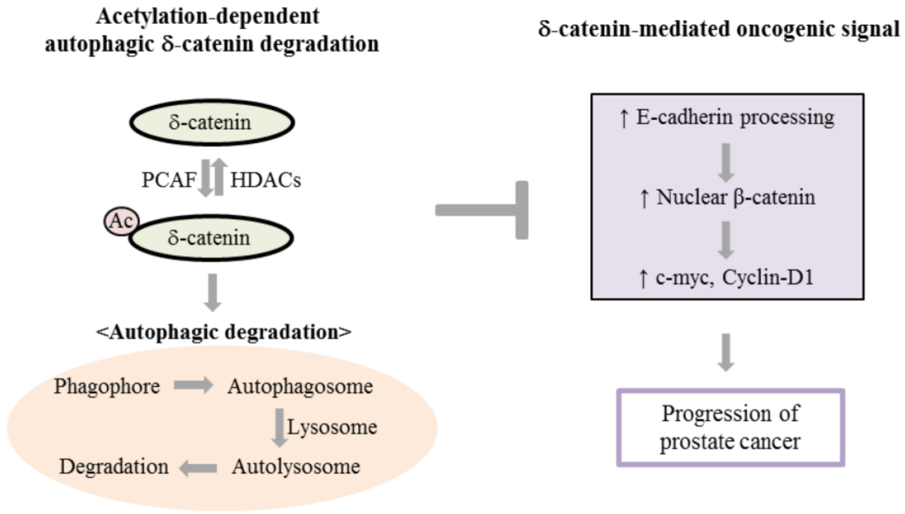

Supplementary Figure 3

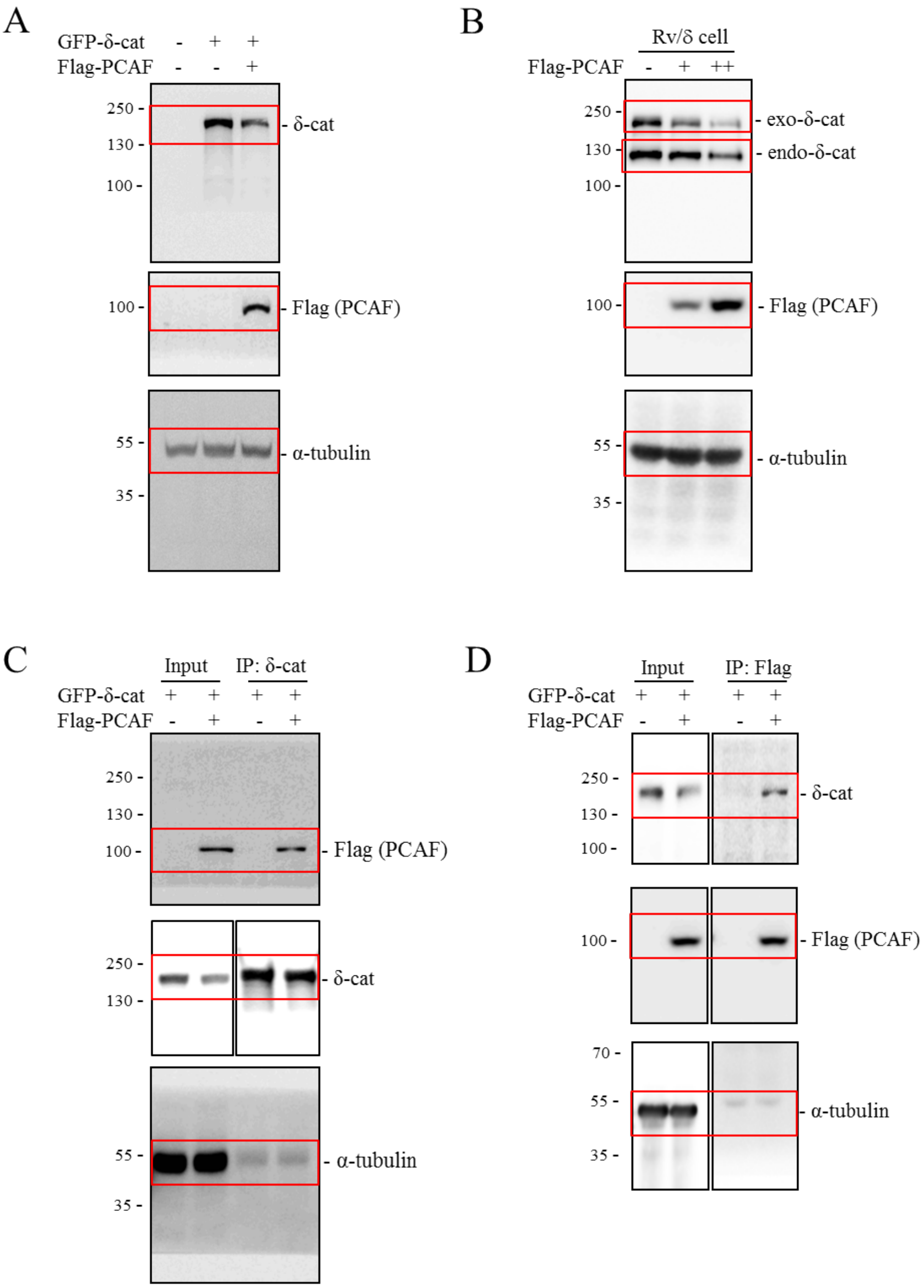

Supplementary Figure 4

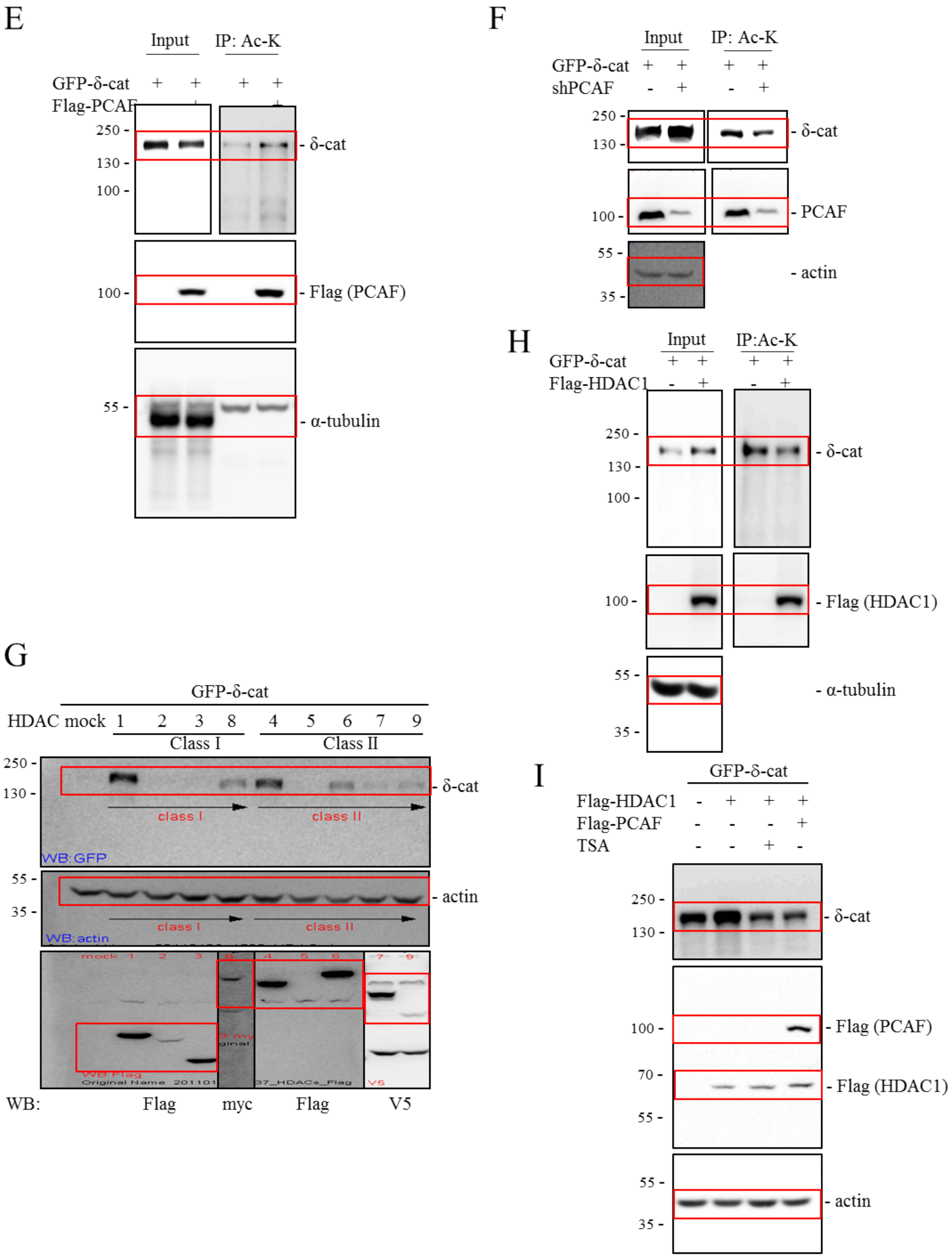

Supplementary Figure 4

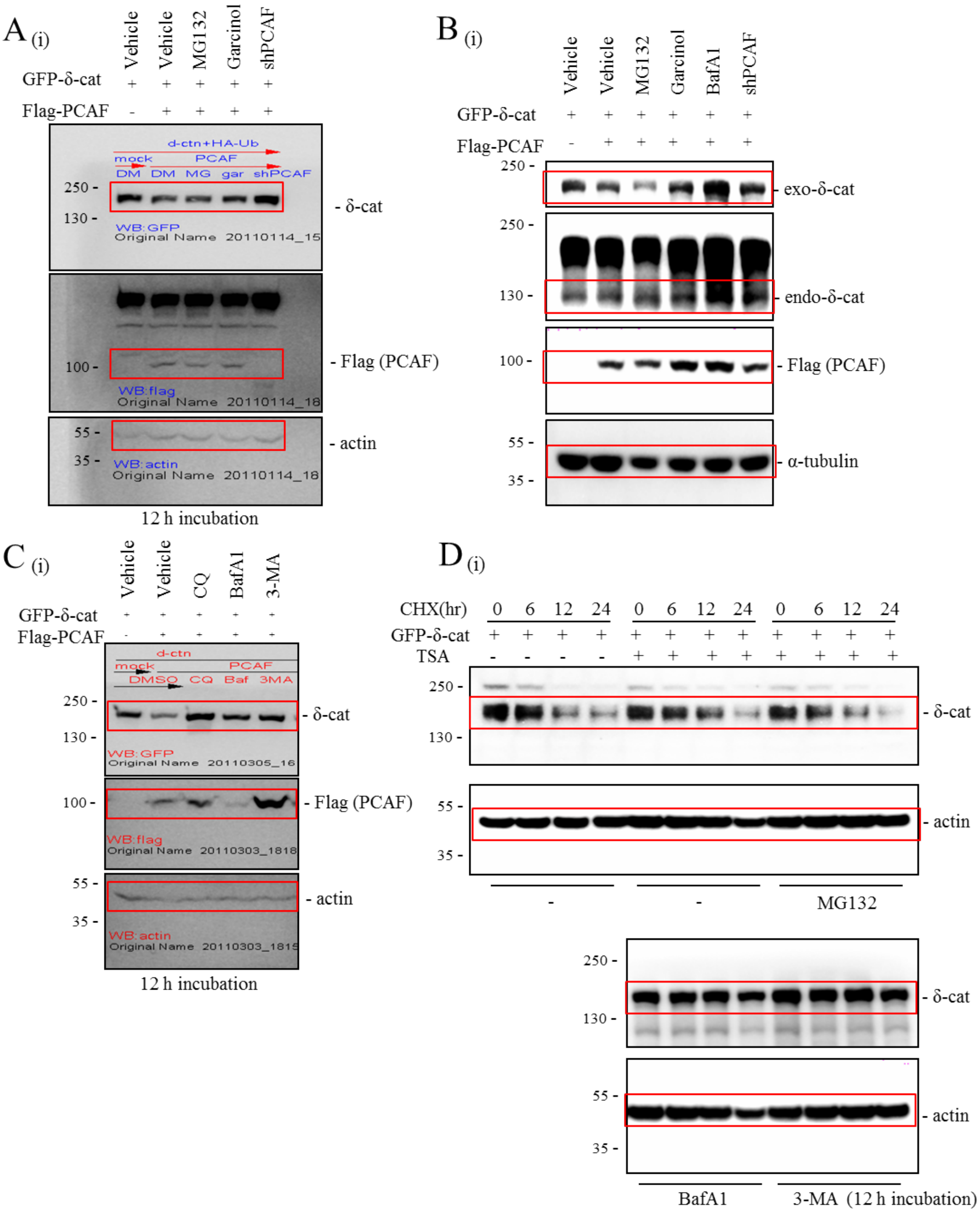

Supplementary Figure 5

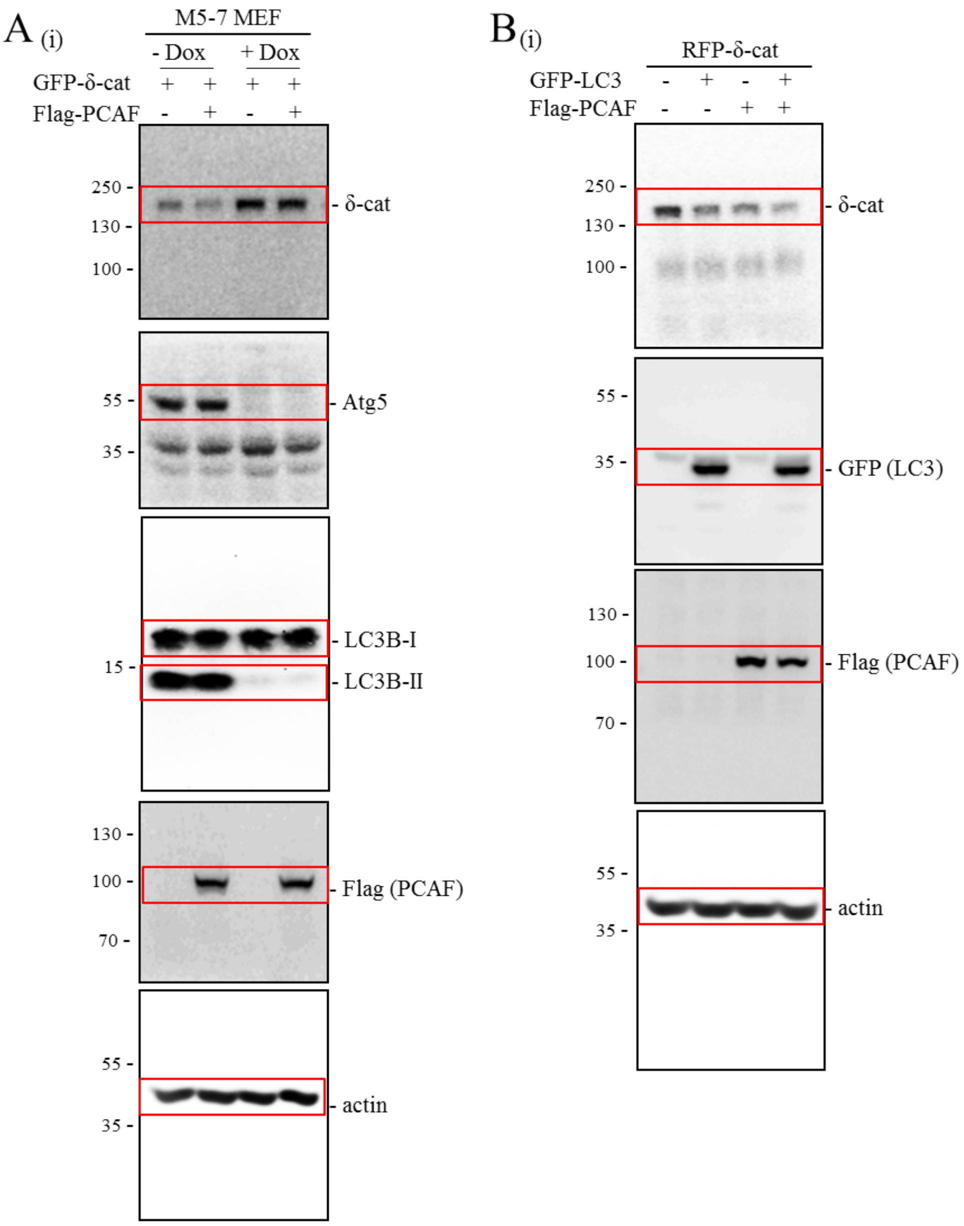

Supplementary Figure 6

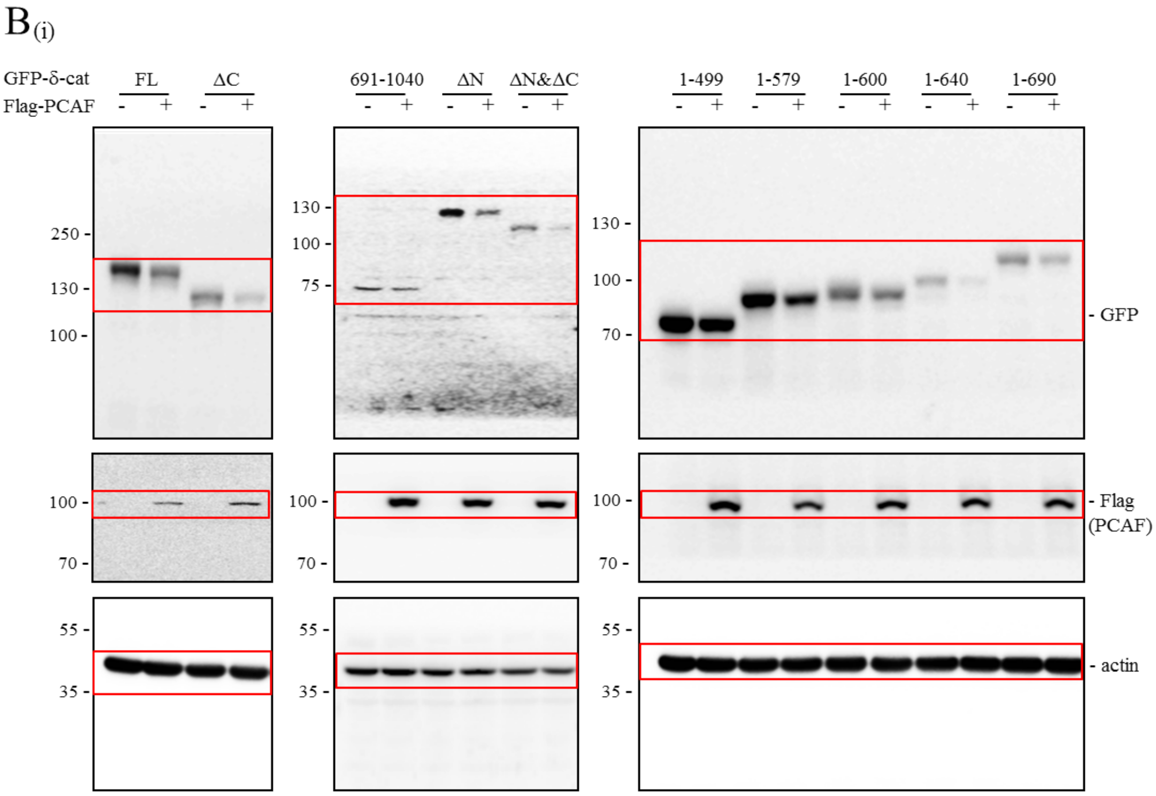

Supplementary Figure 7

B (i)

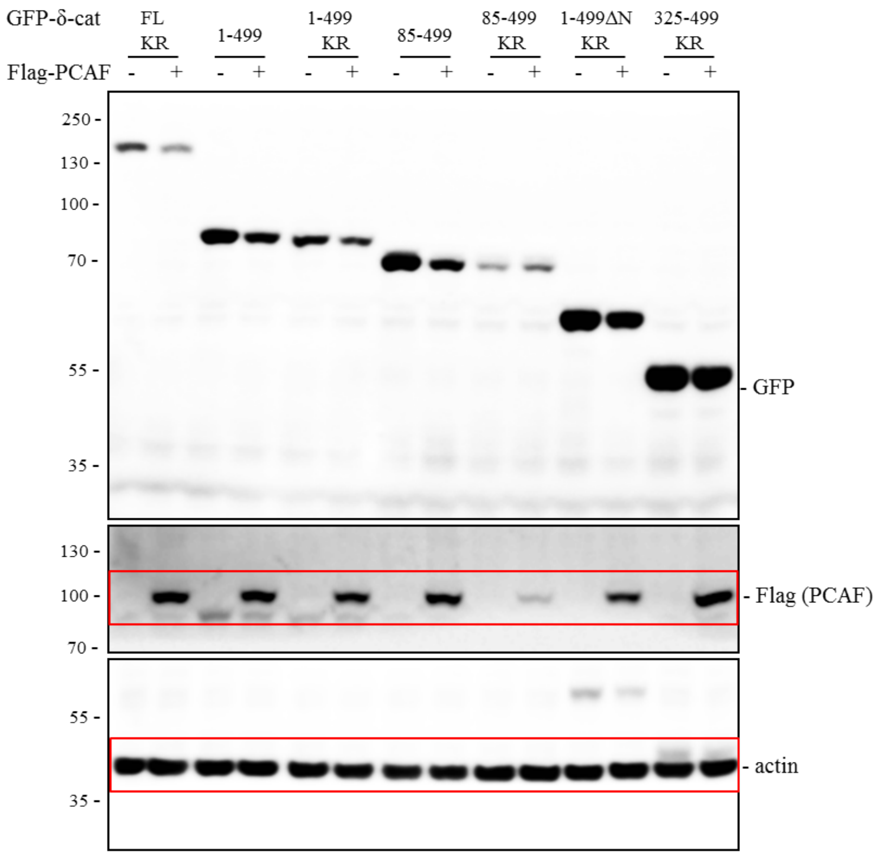

Supplementary Figure 8

C (i)

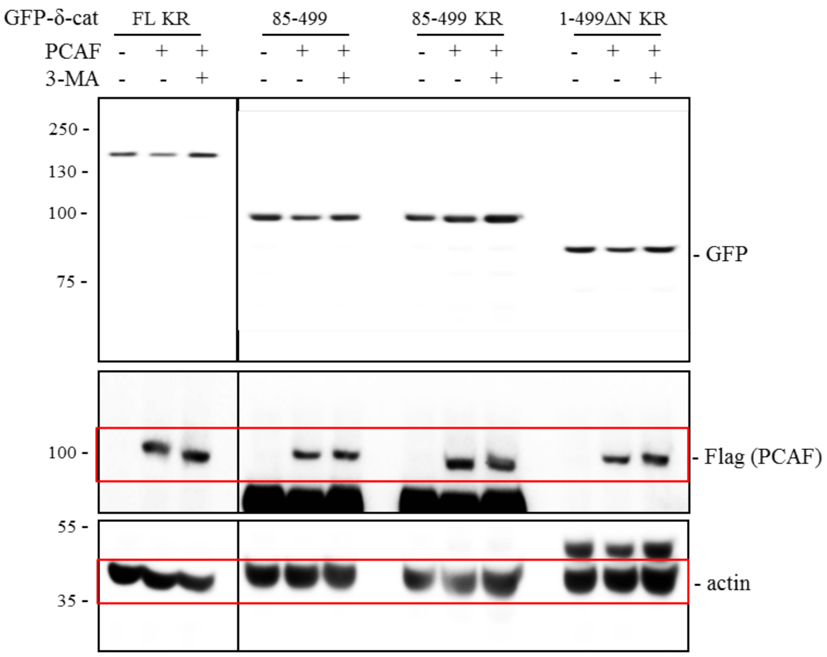

D

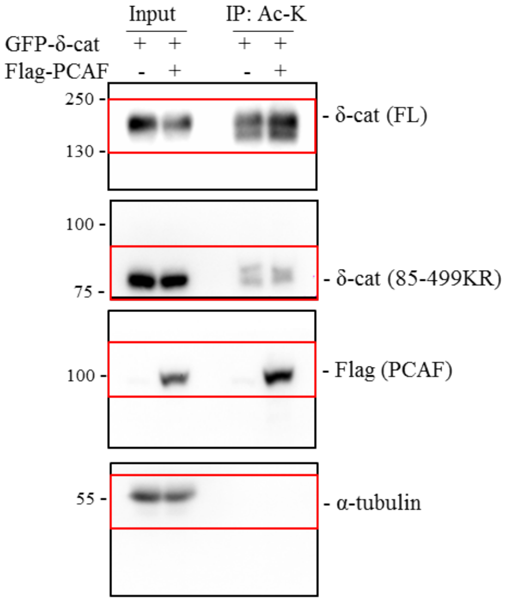

Supplementary Figure 8

A (i)

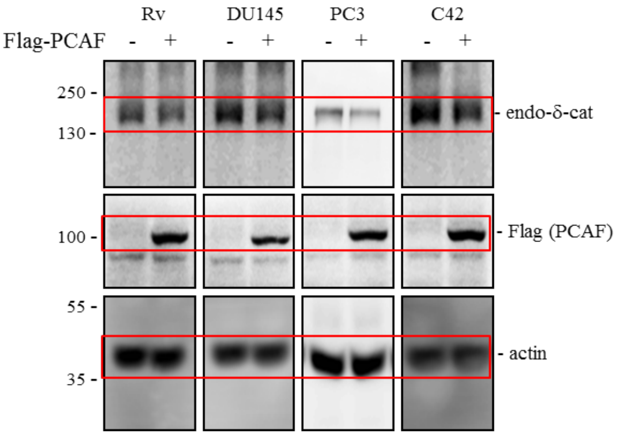

Supplementary Figure 9

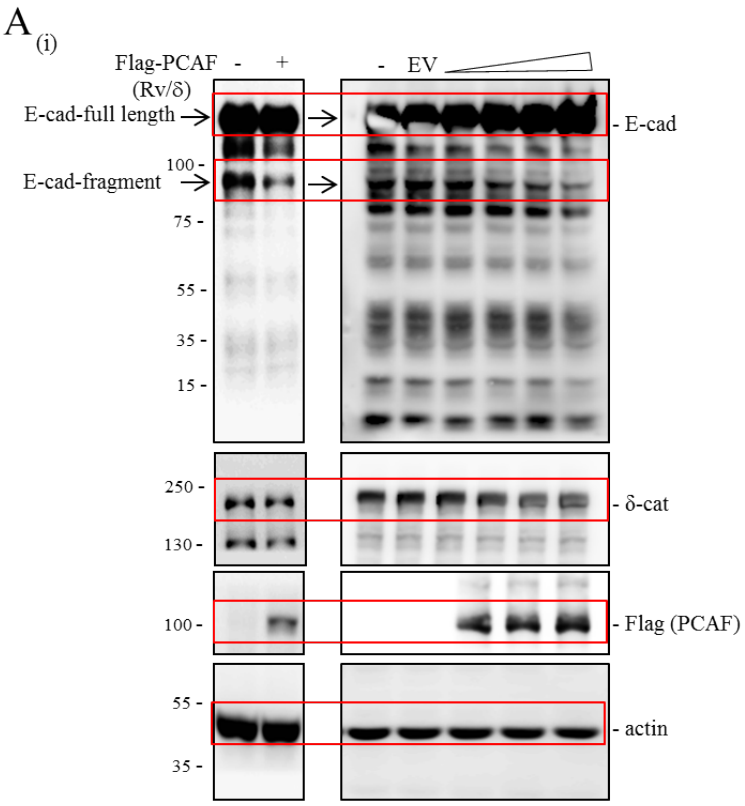

Supplementary Figure 10

B (i)

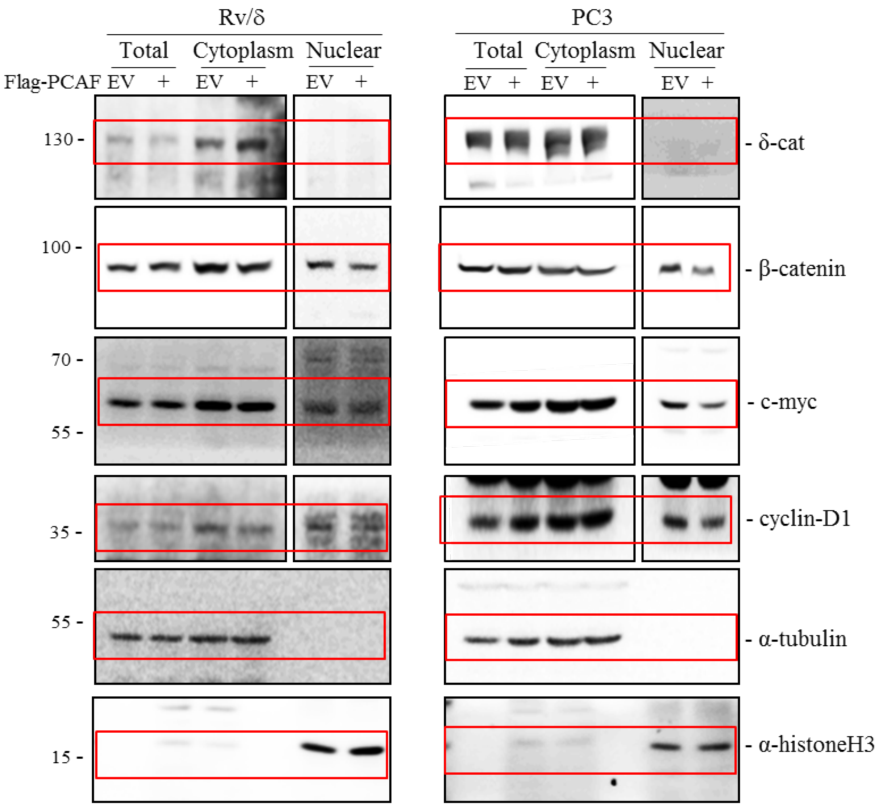

Supplementary Figure 10

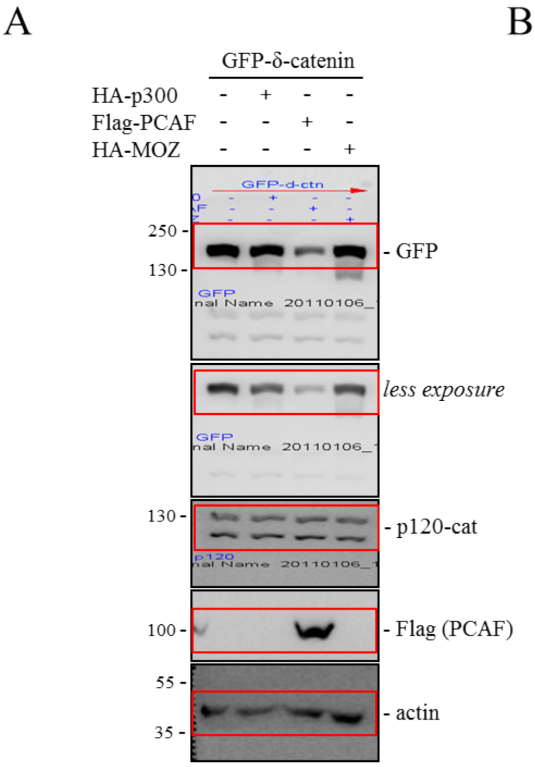

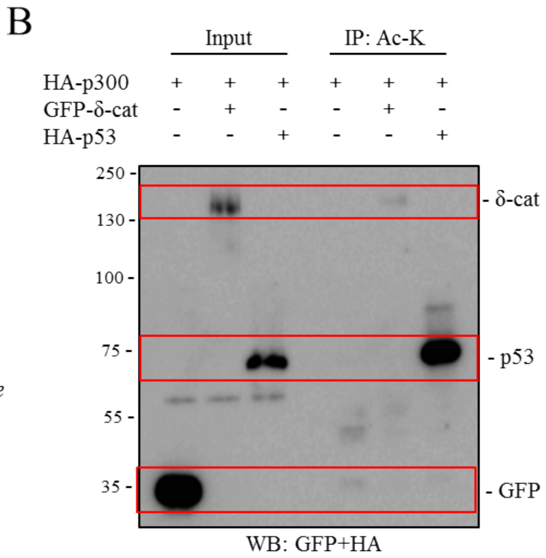

Supplementary Figure 11

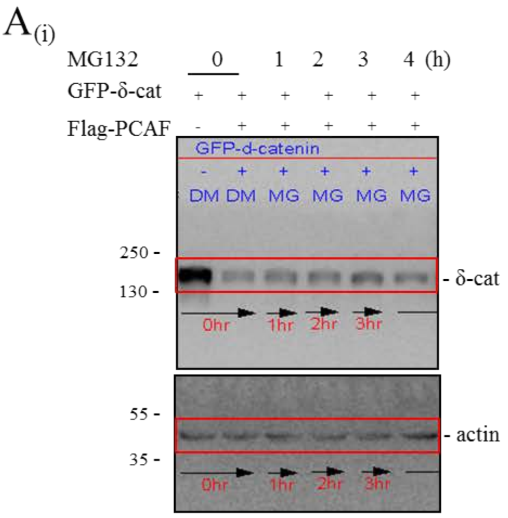

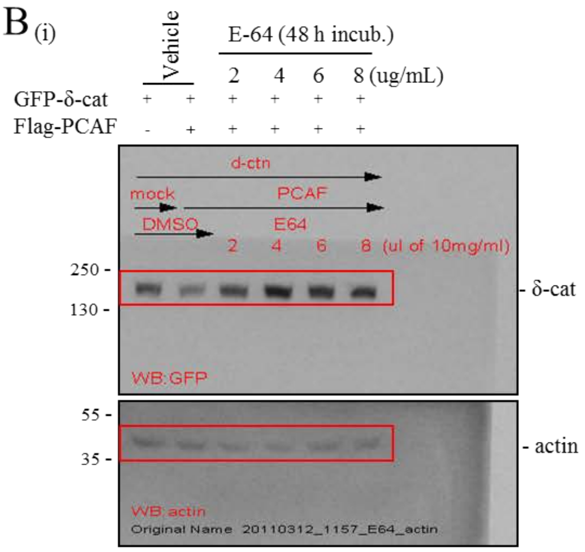

Supplementary Figure 12
